# Carfilzomib in Combination with Bortezomib Enhances Apoptotic Cell Death in B16-F1 Melanoma Cells

**DOI:** 10.3390/biology10020153

**Published:** 2021-02-15

**Authors:** Min Seung Lee, So Hyun Lim, Ah-Ran Yu, Chi Yeon Hwang, Insug Kang, Eui-Ju Yeo

**Affiliations:** 1 Department of Biochemistry, College of Medicine, Gachon University, Incheon 21999, Korea; shine-ms@nate.com (M.S.L.); Dlathgus1@naver.com (S.H.L.); 2 Department of Biomedical Sciences, Graduate School, Kyung Hee University, Seoul 02447, Korea; yoo8088@hanmail.net (A.-R.Y.); clehdrnfl@naver.com (C.Y.H.); 3 Department of Biochemistry and Molecular Biology, School of Medicine, Biomedical Science Institute, Kyung Hee University, Seoul 02447, Korea

**Keywords:** apoptosis, B16-F1 melanoma cells, bortezomib, carfilzomib, proteasome inhibitor

## Abstract

**Simple Summary:**

The incidence rate of metastatic melanoma has been rapidly increasing worldwide and its 5-year survival rate is very low. Due to partial responses, various side effects, and resistance to any known cancer therapeutics, more potent and safer therapeutics are needed to increase the survival rate of patients with melanoma. Since proteasome inhibitors, such as bortezomib and carfilzomib, have been suggested as treatments for various cancers, we investigated their potential for the treatment of melanoma by studying their molecular mechanisms of action in B16-F1 melanoma cells. In this study, we found that both bortezomib and carfilzomib lead to apoptosis via ER stress as well as ROS accumulation and MMP loss in melanoma cells. Bortezomib and carfilzomib synergistically reduced B16-F1 tumor growth in vitro and in a C57BL/6 xenograft mouse model. Therefore, a combination therapy with carfilzomib and bortezomib at submaximal concentrations may reduce their side effects and be beneficial for melanoma treatment.

**Abstract:**

Proteasome inhibitors, such as bortezomib (BZ) and carfilzomib (CFZ), have been suggested as treatments for various cancers. To utilize BZ and/or CFZ as effective therapeutics for treating melanoma, we studied their molecular mechanisms using B16-F1 melanoma cells. Flow cytometry of Annexin V-fluorescein isothiocyanate-labeled cells indicated apoptosis induction by treatment with BZ and CFZ. Apoptosis was evidenced by the activation of various caspases, including caspase 3, 8, 9, and 12. Treatment with BZ and CFZ induced endoplasmic reticulum (ER) stress, as indicated by an increase in eIF2α phosphorylation and the expression of ER stress-associated proteins, including GRP78, ATF6α, ATF4, XBP1, and CCAAT/enhancer-binding protein homologous protein. The effects of CFZ on ER stress and apoptosis were lower than that of BZ. Nevertheless, CFZ and BZ synergistically induced ER stress and apoptosis in B16-F1 cells. Furthermore, the combinational pharmacological interactions of BZ and CFZ against the growth of B16-F1 melanoma cells were assessed by calculating the combination index and dose-reduction index with the CompuSyn software. We found that the combination of CFZ and BZ at submaximal concentrations could obtain dose reduction by exerting synergistic inhibitory effects on cell growth. Moreover, this drug combination reduced tumor growth in C57BL/6 syngeneic mice. Taken together, these results suggest that CFZ in combination with BZ may be a beneficial and potential strategy for melanoma treatment.

## 1. Introduction

Cancer is one of the major health problems worldwide. Over the past centuries, biological and pathological discoveries pertaining to cancers have contributed to the development of an effective chemotherapeutic strategy using cytotoxic anticancer drugs. Subsequently, molecular and cellular biology studies have allowed the development of specific molecular target drugs, such as selective kinase inhibitors and monoclonal antibodies against proteins involved in neoplastic processes [1]. Combinational chemotherapy and targeted therapy using several drugs with different mechanisms of action have been proposed to achieve greater therapeutic efficacy than that by single chemotherapy or targeted therapy in clinical practice [2]. Currently, many researchers are focusing on the development of immune checkpoint inhibitors, immune cell therapies, antitumor vaccines, and other biotechnological drugs. Although these are still in preclinical and clinical trials, the efficacy of some has already been proven in certain types of cancer.

In recent decades, the incidence rate of a metastatic skin cancer called cutaneous melanoma has been rapidly increasing worldwide [3]. It is one of the leading causes of cancer-related mortality [4,5]. In addition to tumor thickness, ulceration, mitotic activity, and solar elastolysis are strong prognostic factors for cutaneous melanoma. Increased exposure to ultraviolet (UV) rays from the sun may be the leading cause of increased cutaneous melanoma incidence [6]. However, the molecular mechanism of UV-induced cutaneous melanoma development is complex [7]. The high UV-dependent mutational loads may be associated with a growing number of genetic alterations, such as activating mutations in oncogenic genes, including *N-ras* and *B-Raf* in the mitogen-activated protein kinase (MAPK) signaling pathway [8,9], and inactivating mutations in tumor suppressor genes, including *p16^Ink4a^/p19^Arf^* and *p53* [10]. Genomic alterations also inactivate *phosphatase and tensin homolog* (*PTEN*), thereby leading to the aberrant activation of the phosphoinositol-3-kinase (PI3K) pathway in melanoma [4]. Furthermore, the tumor microenvironment and immune system interact with melanoma cells, driving their malignant transformation [4].

Although melanoma has a high rate of early diagnosis, its 5-year survival rate is very low (about 15% to 20% at stage IV) due to resistance to any known cancer therapeutics [11]. For treating melanoma, the US Food and Drug Administration (FDA) has approved several immunotherapy drugs that strengthen the immune system defense against melanoma cells and various targeted therapies to inhibit melanoma tumor growth [12], including the oncogenic B-Raf inhibitors vemurafenib [13] and dabrafenib [14] and the MEK inhibitor trametinib [15]. However, melanoma cells with a mutant B-Raf show resistance to most targeted inhibitors [16]. Combined targeted and immune therapies exert durable responses in relatively few patients [17]. Moreover, treatment of *B-Raf^V600^*-mutant melanoma using a B-Raf inhibitor or its combination with a MEK inhibitor typically elicits only partial responses due to the tumor cell-intrinsic reprogramming that attenuates the MAPK dependency [18]. Currently, dacarbazine, an alkylating agent that cross-links DNA resulting in cell cycle arrest and apoptosis, is the only FDA-approved general chemotherapy drug for treating stage IV metastatic melanoma. However, this drug exerts various side effects, such as liver and kidney damage and secondary malignancy induction [19]. Therefore, more potent and safer therapeutics are needed to increase the survival rate of patients with melanoma.

The 26S proteasome plays a role in ubiquitin-dependent degradation of cellular misfolded proteins and consists of two outer ATP-dependent 19S regulatory particles and an inner 20S catalytic core particle. The 20S proteasome is organized as four stacked rings (two outer α-rings and two inner β-rings) of seven distinct subunits each [20,21]. Because inhibition of the proteasome leads to cell growth inhibition and apoptotic cell death in certain tumor-derived cell lines, proteasome inhibitors have been suggested as cancer therapeutics [22,23,24]. Bortezomib (BZ, also known as PS-341 and Velcade^®^) was the first potent in-class proteasome inhibitor that was approved by the FDA and by the European Agency for the Evaluation of Medicinal Products for the treatment of patients with relapsed and/or refractory multiple myeloma (MM) and, later, mantle cell lymphoma [25,26,27,28]. BZ is a synthetic, boronic acid dipeptide, which binds mainly the β5 subunit and, to a lesser extent, the β1 and β2 subunits of the 20S catalytic core, blocking the chymotrypsin-like, caspase-like, and trypsin-like protease activities, respectively [24]. Because the binding of BZ with the β subunits of 20S proteasome forms a tetrahedral transition state intermediate, the reversibility of its binding has been suggested. However, recent crystal structure analyses have demonstrated that the covalent binding of boron atom to the nucleophilic oxygen of Thr^1^-γOH and the formation of additional tight hydrogen bonds between the boronate hydroxyl groups, the amine atoms of Thr^1^ and/or Gly^47^, and a catalytic water in the active site enhance the stability of tetrahedral intermediate, resulting in practical irreversibility and high affinity binding of BZ to the proteasome [29,30].

BZ is known to induce substantial off-target activities and causes various side effects, such as peripheral neuropathy, myelosuppression, and cardiac and skeletal toxicities, leading to dose modulation or early discontinuation of the therapy [31]. However, BZ in combination with other drugs for chemosensitization and synergy, immunomodulators such as lenalidomide or thalidomide, and corticosteroids such as dexamethasone or prednisone is commonly used as a first-line agent for the treatment of MM patients [32,33,34]. BZ in combination with other agents has also shown significant preclinical and clinical activity in other types of malignancies, including non-Hodgkin’s lymphoma [35], acute myeloid leukemia [36], advanced renal cell carcinoma [37], and metastatic melanomas [38].

Carfilzomib (CFZ, also known as PR-171, Kyprolis^®^) is one of the second generation proteasome inhibitors with improved efficacy and minimal off-target adverse effects, being approved by the FDA and utilized for the treatment of relapsed and refractory MM as a single agent [39,40,41] or in combination with other drugs [33,42]. CFZ is an epoxomicin derivative with a chemical structure of tetrapeptide epoxyketone that irreversibly binds to the β5 subunit and inhibits its chymotrypsin-like activity of the 20S catalytic core [43,44,45]. A recent refinement of X-ray crystal structures has suggested that the efficacy of epoxyketone inhibitors depends on their ability to form a seven-membered ring structure, a 1,4-oxazepane, via nucleophilic attack of the epoxide β carbon by the amine of the active site Thr^1^ [30]. This CFZ-selective mechanism of action may be responsible for much improved safety profiles [46]. Compared to BZ, CFZ has a lower risk of neurotoxicity and a better effect in extending overall survival of the relapsed patients [47]. Nevertheless, CFZ has higher risks than BZ in some other events, such as cardiovascular toxicity [48,49] and renal toxicity [50]. However, these toxicities can be modulated by co-administration with other drugs, such as metformin [49]. Therefore, the use of CFZ alone or in combination with other drugs has recently expanded to the treatment of other solid cancers, such as non-small cell lung cancers [51], breast cancer [52], and neuroblastoma [53]. Because the limitations of existing proteasome inhibitor drugs still remain unresolved, researchers have been working on strategies to overcome their limitations as well as to develop new next-generation proteasome inhibitor drugs [46].

The proposed action mechanisms of cellular toxicity induced by proteasome inhibitors include accumulation of misfolded proteins, which leads to the activation of unfolded protein responses (UPR) [54]. The UPR is mediated by three endoplasmic reticulum (ER) transmembrane receptors, including inositol requiring kinase 1α (IRE1α), activating transcription factor 6 (ATF6), and double-stranded RNA-activated protein kinase-like ER kinase (PERK) [55]. The UPR produces activated transcription factors, such as XBP-1 and cleaved ATF6 and ATF4, resulting in upregulation of ER stress-associated proteins, such as GRP94, GRP78, and CCAAT-enhancer-binding protein homologous protein (CHOP). If the UPR could not regenerate folding homeostasis, persistent UPR causes ER stress and apoptosis [56]. ER stress is closely associated with reactive oxygen species (ROS) production and mitochondrial dysfunctions in cancer cells [53,57]. Moreover, the cytotoxic effects of proteasome inhibitors have also included the inhibition of nuclear factor-κB (NF-κB) pathway, activation of the c-Jun NH2-terminal kinase (JNK) pathway, phosphorylation of p53, and cell cycle arrest at G1 via the induction of cyclin-dependent kinase inhibitors, such as p21 and p27 [58,59,60]. Because the management of these molecular mechanisms is important to improve their therapeutic outcomes and to reduce the side effects, further studies on the molecular mechanisms are needed.

To better understand the effects of proteasome inhibitors in melanoma treatment, in this study, we compared the molecular mechanisms of two proteasome inhibitors, BZ and CFZ, on the ER stress and apoptosis of B16-F1 melanoma cells. We also examined the effect of a combination therapy with BZ and CFZ in C57BL/6 syngeneic xenograft model mice.

## 2. Results

### 2.1. BZ and CFZ Induce Apoptosis in Various Types of Melanoma Cells Including B16-F1 Cells

**Figure 1:**
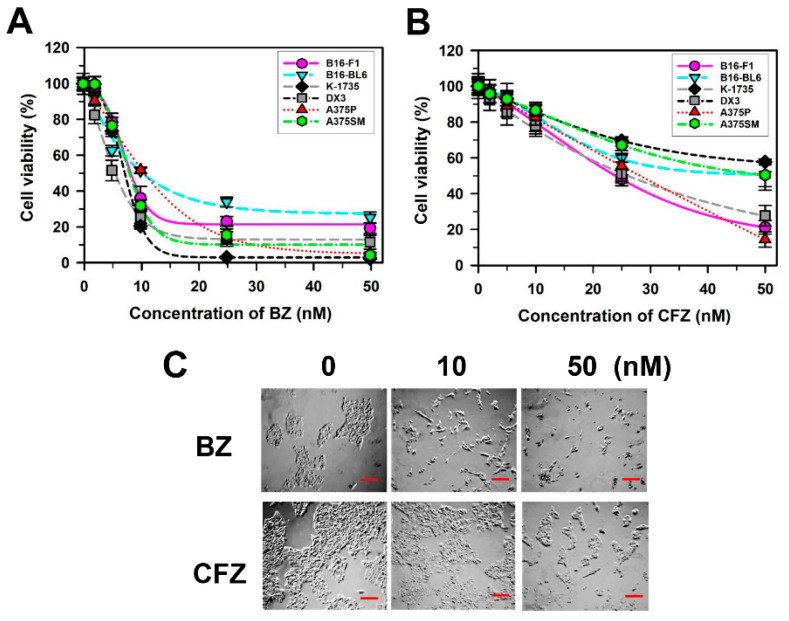
Effect of BZ and CFZ on cell viability of various melanoma cells. (**A**,**B**) B16-F1, B16-BL6, K-1735, DX3, A375P, and A375SM were treated with a vehicle or various concentrations of BZ (**A**) or CFZ (**B**) for 2 days in the culture medium. Cell viability was assessed by MTT assay and the percent viabilities were calculated (*n* = 12 for total replicates). The regression fit curves and dots corresponding to the drug concentration as average ± standard deviation are plotted using Sigma Plot. (**C**) The morphological changes were observed by an inverted microscope after treatment with 10 and 50 nM BZ or CFZ for 2 days, and the 100× magnified photos with 100 µm scale bar are shown.

The effects of BZ and CFZ on cell viability were examined by an assay with dimethylthiazol-2-yl)-2,5-diphenyltetrazolium bromide (MTT assay) in spontaneous and metastatic murine melanoma cell line B16-F1 as well as another B16-derived murine melanoma cell line with higher metastatic potential B16-BL6; carcinogen-induced murine melanoma cell line K-1735; and the DX3, A375P (lowly metastatic), and A375SM (highly metastatic) human melanoma cell lines. We observed that BZ and CFZ reduced the viability of various types of melanoma cells in a concentration-dependent manner (Figure 1A,B). Moreover, these drugs exerted varying activities against the various melanoma cell lines. CFZ was less cytotoxic than BZ towards the melanoma cells (Figure 1A,B), which was confirmed by the higher IC_50_ values for CFZ than that for BZ (Table 1). The murine B16 melanoma cell line is one of the most widely used cancer models [61]. In addition, B16-F1 cells can engraft in syngeneic and immunocompetent C57BL/6 mice, which would be very useful for further analyses on melanoma. Therefore, B16-F1 cells were chosen to determine the molecular mechanisms associated with anticancer effects of BZ and CFZ. The IC_50_ values were 8.1 nM and 23.9 nM for BZ and CFZ, respectively (Table 1), and their differential effects on B16-F1 cell morphology and death were observed under an inverted microscope (Figure 1C).

**Figure 2:**
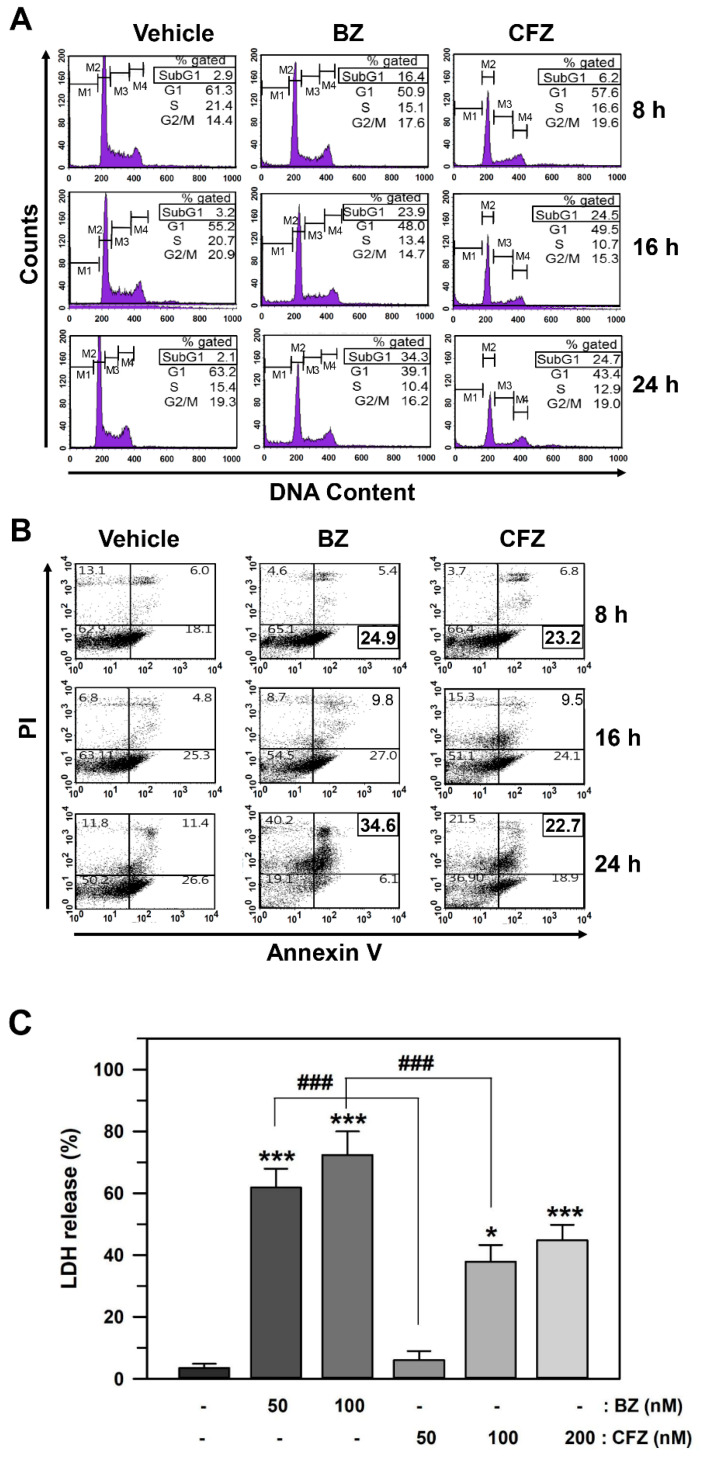
Effect of BZ and CFZ on apoptosis in B16-F1 cells. (**A**,**B**) Cells were treated with vehicle or 50 nM BZ or 100 nM CFZ in the culture medium for the indicated times. Ethanol-fixed cells were stained with propidium idodide (PI) for DNA fragmentation detection (**A**) and intact cells were double-stained with PI and annexin V-FITC for apoptosis detection (**B**). The fluorescence was evaluated by flow cytometry and the percentage of cells was calculated (*n* = 9 for total replicates). The average values are shown in the upper corner of each area. (**C**) The cytotoxicity was determined by LDH release assay and the percent cytotoxicity is plotted as means ± standard deviations (*n* = 12 for total replicates). The statistically significant difference between groups was determined by one-way ANOVA followed by Dunnett’s T3 post hoc test. * *p* < 0.05 and *** *p* < 0.001 compared with the vehicle-treated control. ^###^ *p* < 0.001 compared between BZ and CFZ treatment groups at equal concentration. (**A**–**C**) Cells were treated with various concentrations of BZ or CFZ for 24 h in 2% FBS/DMEM or 10% FBS/DMEM, or with 100 nM BZ or CFZ for the indicated times in 2% FBS/DMEM (F). Total cell extracts were analyzed by Western blotting with antibodies against the cleaved forms of caspases (Cas 3, 8, 9, and 12) and β-actin. The abbreviation (**C**) after the name of caspases stands for “cleaved”. The band densities were normalized against β-actin (*n* = 6 for total replicates) and the fold changes compared to that of vehicle-treated control (0 nM in **A** and **B**, −/− at 4 h in **C**) are written under each band.

Next, we determined whether BZ and CFZ reduced cell viability via cell cycle arrest and/or apoptotic cell death in B16-F1 cells. Cell cycle arrest and DNA fragmentation were examined using flow cytometry for DNA content analysis. Cells in the subG1 fraction were significantly increased from 2.1% to 34.3% and 24.7% at 24 h after treatment with 50 nM BZ and 100 nM CFZ, respectively (Figure 2A). These results suggested that BZ and CFZ could induce DNA fragmentation in B16-F1 cells. In contrast, the percentage of cells in the G1, S, and G2/M fractions was not significantly altered, indicating that cell cycle arrest might not be a major causative factor for reduction in viability. For detecting apoptosis, the annexin V-Fluorescein isothiocyanate (FITC) and propidium iodide (PI) double staining experiment was performed. Treatment with 50 nM BZ and 100 nM CFZ increased the number of early apoptotic cells (lower right area) from 18.1% to 24.9% and 23.2%, respectively, at 8 h and that of late apoptotic cells (upper right area) from 11.4% to 34.6% and 22.7%, respectively, at 24 h (Figure 2B). A lactate dehydrogenase (LDH) release assay was performed to compare their cytotoxic effects. There was a statistically significant difference between the groups as determined by the one-way ANOVA (*F*(5,42) = 240.901, *p* < 10^–3^). Dunnett’s T3 post hoc test demonstrated that BZ exerted a more statistically significant cytotoxic effect than CFZ at 24 h after treatment with equal concentrations (Figure 2C).

BZ- and CFZ-induced apoptosis were confirmed by the presence of cleaved caspase 3, 8, 9, and 12. Western blotting revealed that the overall effect of BZ on caspase activation was stronger than that of CFZ at the same concentration (Figure 2D,E, Figures S1 and S2). In addition, the high level (10% compared to 2%) of fetal bovine serum (FBS) in the Dulbecco’s modified Eagle’s media (DMEM) reduced the caspase activation signals in BZ- and CFZ-treated cells (Figure 2D,E, respectively). Time-dependent Western blot analyses with cells cultured in 2% FBS also showed that the activation of caspase 3 was evident at 24 h after treatment with 100 nM BZ and CFZ (Figure 2F, Figure S3). BZ treatment began to activate caspase 8, 9, and 12 at 16 h and later enhanced their activation at 24 h. In contrast, CFZ treatment slightly activated caspase 9 and 12 at 16 h but caspase 8 at 24 h. Collectively these results suggested that both BZ and CFZ could induce apoptosis via the activation of canonical caspases implicated in the intrinsic and extrinsic pathways, but their contributions were slightly different, as observed in the BZ- and CFZ-treated cells.

### 2.2. BZ and CFZ Induce Apoptosis via ER Stress in B16-F1 Cells

**Figure 3:**
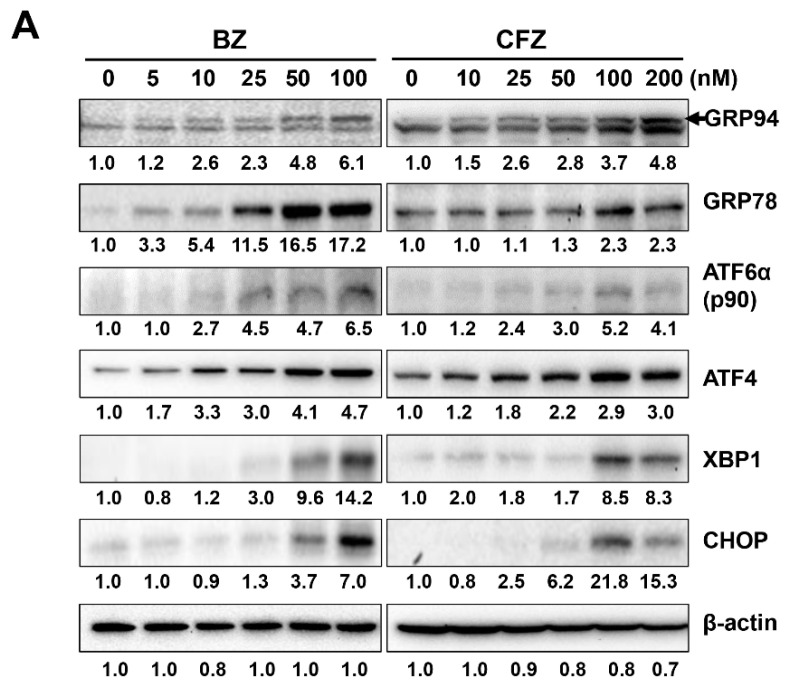

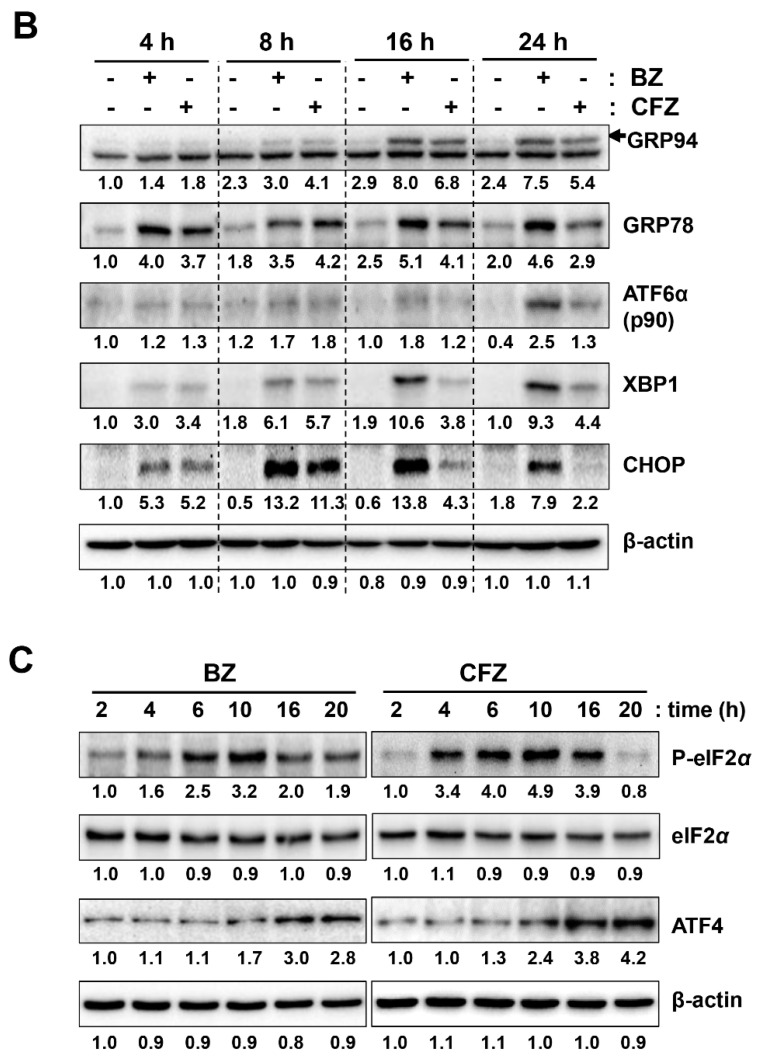
Effect of BZ and CFZ on ER stress-associated proteins in B16-F1 cells. (**A**–**C**) Cells were treated with vehicle or various concentrations of BZ or CFZ for 24 h (**A**), or with vehicle or 100 nM BZ or CFZ for the indicated times in 2% FBS/DMEM (**B**,**C**). Cell lysates were analyzed by Western blotting with antibodies against ER stress-associated proteins (GRP94, GRP78, p90 ATF6α, ATF4, XBP1, CHOP, *p*-eIF2α, and eIF2α) and β-actin. The band densities were normalized against β-actin (*n* = 6 for total replicates) and the fold changes compared to that of vehicle-treated control (0 nM in **A**, −/− at 4 h in **B**) or that of early time point (2 h in **C**) are written under each band.

BZ and CFZ activate caspase 12, which is related to ER stress [62]. To determine ER stress induction, B16-F1 cells were treated with various concentrations of BZ and CFZ for 24 h, and the expression of ER stress-associated proteins, such as GRP94, GRP78, soluble XBP1, and CHOP, and the cleavage of ATF6α was examined by Western blot analysis. The concentration-dependent Western blotting data clearly showed that the levels of some of the ER stress markers, such as GRP78, ATF6α, and XBP1, were greater in the BZ-treated cells than in the CFZ-treated cells at the same concentration (Figure 3A, Figure S4). Moreover, levels of these ER stress-associated proteins increased with BZ and CFZ treatment in a time-dependent manner (Figure 3B). Interestingly, BZ-induced CHOP expression reached its peak at 8–16 h and then decreased at 24 h, whereas CFZ-induced CHOP expression reached its peak at 8 h and then gradually reduced from 16 to 24 h (Figure 3B, Figure S5). The time-dependent effect of BZ and CFZ on the initial UPR was also examined by measuring the levels of phosphorylated eIF2α and ATF4. Both BZ and CFZ increased the levels of phosphorylated eIF2α, achieving the maximum level at 10 h (Figure 3C, Figure S6), and resulted in inhibition of eIF2α activity and protein synthesis. The expression of ATF4 gradually enhanced from 10 to 20 h after dephosphorylation of eIF2α. Collectively, our data showed ER stress upon BZ and CFZ treatment and suggested that it may be involved in the apoptotic death of B16F1 cells.

### 2.3. BZ and CFZ Increase ROS Accumulation and Mitochondrial Membrane Potential Loss in B16-F1 Cells

**Figure 4:**
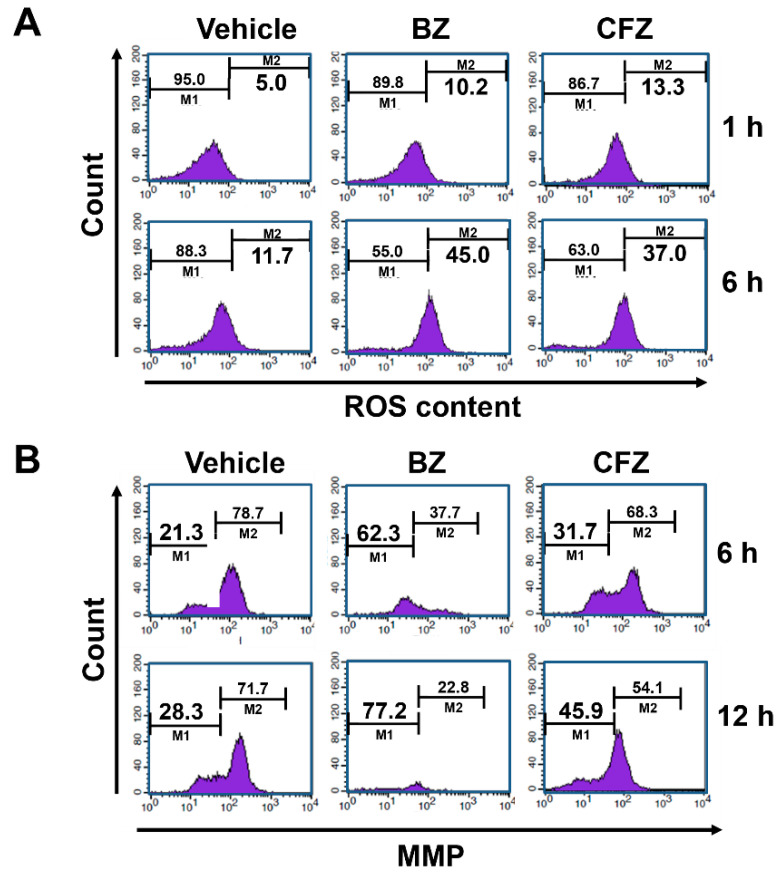
Effect of BZ and CFZ on ROS accumulation and MMP loss in B16-F1 cells. Cells were treated with vehicle or 100 nM BZ or CFZ for the indicated times in 2% FBS/DMEM. Cells were stained with DCFH-DA for ROS detection (**A**) or DiOC_6_ for MMP detection (**B**), and then analyzed by flow cytometry. The percent ROS accumulation and MMP loss were calculated from the fluorescence data and are written in the right M2 and left M1 area, respectively (*n* = 9 for total replicates).

The involvement of ROS in ER stress-induced apoptosis has been previously demonstrated [63]. To determine whether BZ and CFZ could induce ROS accumulation, B16-F1 cells were treated with 100 nM BZ or CFZ, and cellular ROS levels were measured using flow cytometry after treatment with 2′,7′-dichlorodihydrofluorescein diacetate (DCFH-DA). BZ and CFZ treatment increased ROS levels from 11.7% to 45.0% and 37.0%, respectively, at 6 h (Figure 4A). To determine if mitochondrial dysfunction is implicated in BZ- and CFZ-induced apoptosis, mitochondrial membrane potential (MMP) was analyzed by flow cytometry after staining with 3,3′-dihexyloxacarbocyanine iodide (DiOC_6_). The data showed that MMP loss was enhanced from 28.3% to 77.2% and 45.9% after 12 h of treatment with 100 nM BZ and CFZ, respectively (Figure 4B).

**Figure 5:**
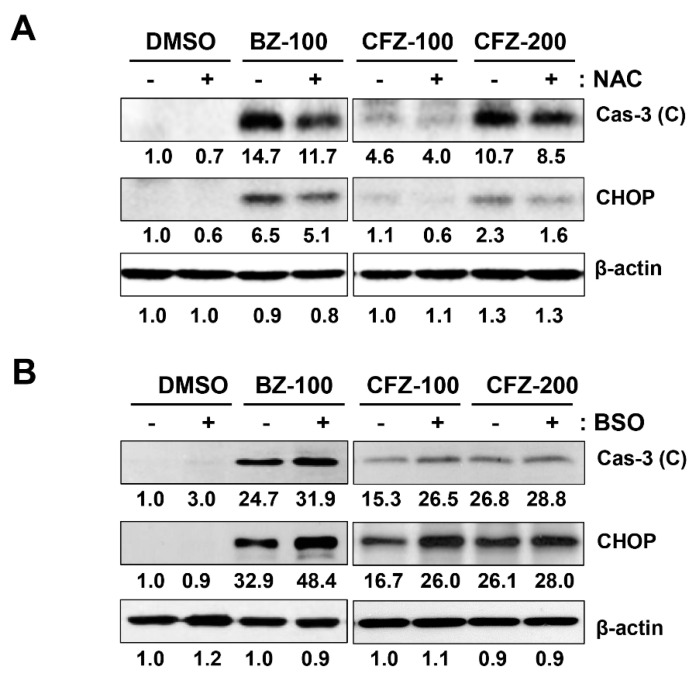

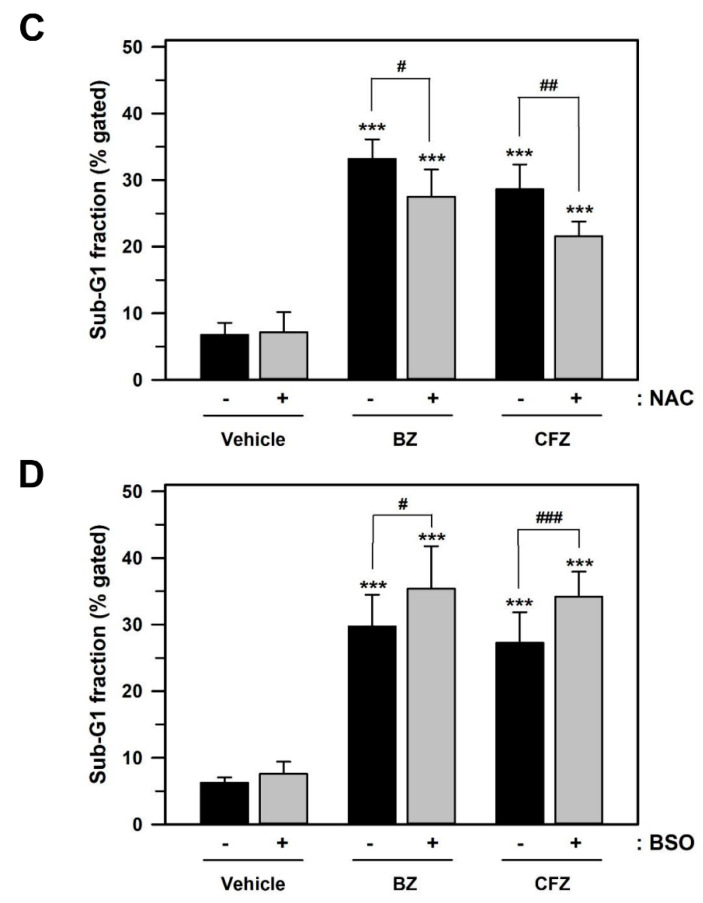
Effect of NAC and BSO on BZ- and CFZ-induced apoptosis and ER stress in B16-F1 cells. (**A**,**B**) Cells were pretreated with 5 mM NAC for 1 h (**A**) or 100 µM BSO (**B**) for 24 h and treated with a vehicle, 100 nM BZ or 100–200 nM CFZ for 24 h in 2% FBS/DMEM. Cell lysates were analyzed by Western blotting with antibodies against cleaved caspase 3, CHOP, and β-actin. The band densities were normalized against β-actin, and the fold changes compared to that in vehicle-treated control are indicated below each band. (**C**,**D**) Cells were pretreated with either NAC (**C**) or BSO (**D**) and then treated with vehicle, 50 nM BZ, or 100 nM CFZ in 2% FBS/DMEM. Ethanol-fixed cells were stained with PI and the gated sub-G1 fraction was determined by flow cytometry and is plotted as means ± standard deviations. (**C**,**D**) Cells were pretreated with NAC (**C**) or BSO (**D**), and then treated with vehicle, 5–20 nM BZ, or CFZ for 2 days in the culture medium. The viability of NAC- or BSO-pretreated cells was determined by MTT assay, and the percent cell viabilities are plotted as means ± standard deviations (*n* = 12 for total replicates). Significant difference between groups was determined by one-way ANOVA followed by Dunnett’s T3 post hoc tests. *** *p* < 0.001 compared with the vehicle-treated control cells. ^#^ *p* < 0.05, ^##^ *p* < 0.01 and ^###^ *p* < 0.001 compared with BZ or CFZ treatment alone without NAC or BSO pretreatment.

To confirm that ROS are involved in BZ- and CFZ-induced ER stress and apoptosis in B16-F1 cells, cells were preincubated with a chemical antioxidant N-acetyl cysteine (NAC) for 1 h, followed by treatment with BZ or CFZ for 24 h. Since glutathione plays a major role in cellular defense against oxidative stress, we pretreated cells with the most commonly used γ-glutamylcysteine synthetase inhibitor buthionine sulfoximine (BSO), which would reduce the levels of cellular oxidant scavenger glutathione and thus, amplify oxidative stress [64]. We found that NAC pretreatment partially reduced BZ- and CFZ-induced caspase 3 activation and CHOP expression (Figure 5A, Figure S7), but treatment with BSO increased the levels of cleaved caspase 3 and CHOP (Figure 5B, Figure S8). Furthermore, the sub-G1 fraction was assessed by flow cytometry to verify the differential effects of NAC and BSO on BZ/CFZ-induced cell death. One-way ANOVA revealed a statistically significant difference in the sub-G1 fractions between groups with NAC pretreatment (*F*(5, 54) = 136.964, *p* < 10^−3^) or BSO pretreatment (*F*(5, 174) = 298.097, *p* < 10^−3^). Dunnett’s T3 post hoc test demonstrated that NAC pretreatment significantly reduced the number of cells in the sub-G1 fraction (Figure 5C), but BSO pretreatment increased their number (Figure 5D), indicating that NAC prevents and BSO enhances apoptotic cell death. A cell viability assay was performed to verify the differential effects of NAC and BSO. One-way ANOVA revealed a statistically significant difference between groups with NAC pretreatment (*F*(13, 84) = 220.011, *p* < 10^−3^) or BSO pretreatment (*F*(13, 210) = 529.042, *p* < 10^−3^). Dunnett’s T3 post hoc test demonstrated that NAC exerted a statistically significant protective effect on B16-F1 cells treated with 10–20 nM BZ or 20 nM CFZ (Figure 5E), but BSO exerted a significant cytotoxic effect on B16-F1 cells treated with 5–10 nM BZ or 5–20 nM CFZ (Figure 5F). Taken together, these findings suggested that ROS and MMP loss were the causative factors for BZ- and CFZ-induced ER stress and apoptosis in B16-F1 cells.

### 2.4. BZ and CFZ Synergistically Induce ER Stress and Apoptosis in B16-F1 Cells

The two drugs have the most important features: (i) BZ has high efficacy and exerts various cytotoxic side effects, including peripheral neurotoxicity [65], and (ii) CFZ has low toxicity and is a broad spectrum drug [41]. Therefore, the combination of the two drugs would have favorable consequences if they were synergistic. Even if they only show an additive effect, it would be beneficial to reduce the dosage of cytotoxic BZ.

**Figure 6:**
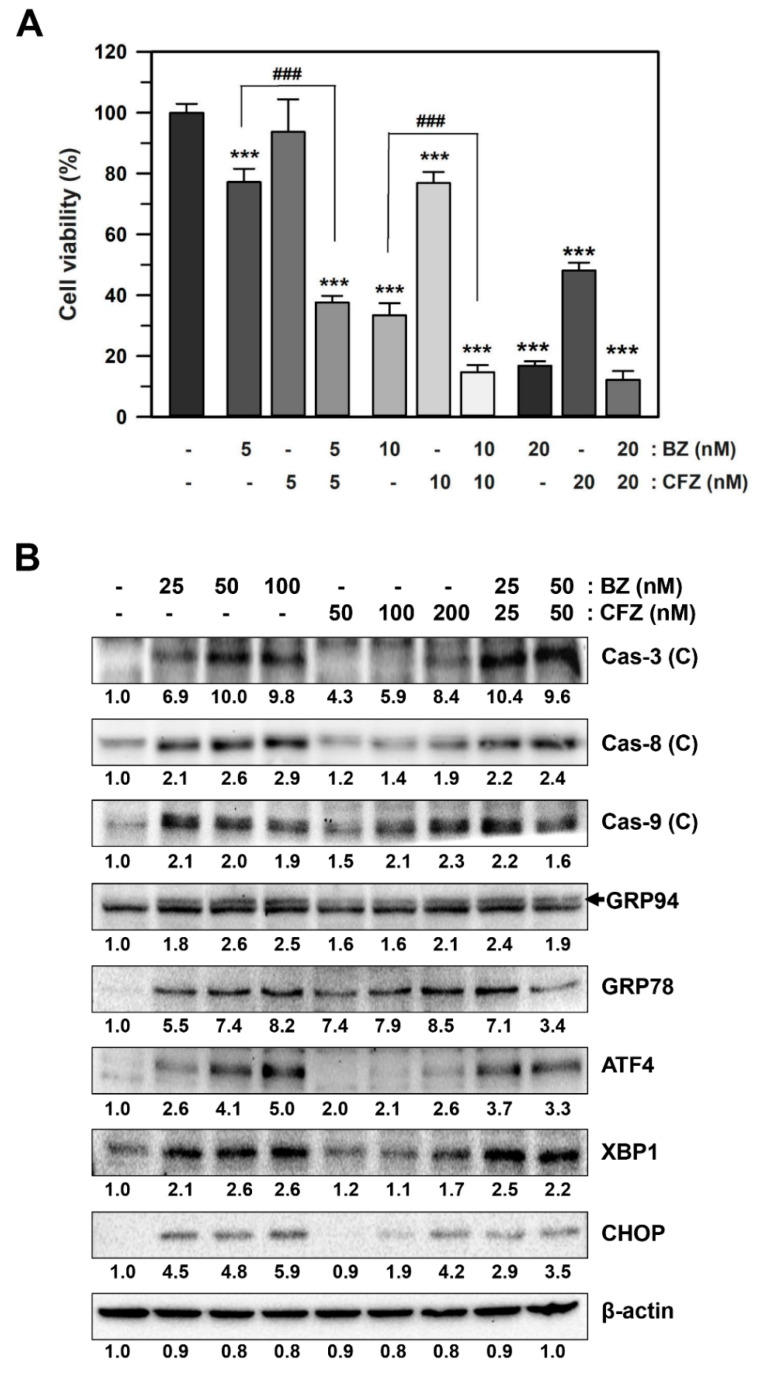
Additive effects of BZ and CFZ on ER stress and apoptosis in B16-F1 cells. (**A**) Cells were treated with BZ or CFZ alone, or combinations of BZ and CFZ for 2 days. Cell viability was determined by MTT assay and the percent cell viabilities are plotted as means ± standard deviations (*n* = 12 for total replicates). The statistically significant difference between groups was determined by one-way ANOVA followed by Dunnett’s T3 post hoc tests. *** *p* < 0.001 compared with the vehicle-treated control and ^###^ *p* < 0.001 compared with 5 or 10 nM BZ alone. (**B**) Cells were treated with BZ or CFZ alone, or combinations of BZ and CFZ for 24 h in 2% FBS/DMEM. Total cell extracts were analyzed by Western blotting with antibodies against cleaved caspases, ER stress-associated proteins, and β-actin. The band densities were normalized against β-actin (*n* = 6 for total replicates), and the fold changes compared to that of vehicle-treated control (–/–) are written under each band.

To test our hypothesis, the effect of the combination of BZ and CFZ on the viability of B16-F1 cells was examined. There was a statistically significant difference between the treatment groups as determined by one-way ANOVA (*F*(9, 70) = 962.221, *p* < 10^−3^), followed by Dunnett’s T3 post hoc test. Although CFZ alone at 5 nM did not exert any significant effect on cell viability, combinational treatment of 5 nM CFZ with 5 nM BZ showed statistically significant (*p* < 0.001) enhancement of BZ-induced cytotoxicity in B16-F1 cells (Figure 6A). Furthermore, co-treatment with BZ and CFZ (5 + 5, 10 + 10, and 20 + 20 nM at a constant ratio of 1:1) resulted in a stronger inhibition of cell viability than that by treatment with either of the drugs alone.

To assess the synergism in these drug combinations in vitro, we calculated the combination index (CI) and dose-reduction index (DRI) using a computer software CompuSyn (ComboSyn Inc.), which is based on the Chou-Talalay method for drug combination [66,67]. Based on these algorithms, CI < 1, CI = 1, and CI > 1 indicate synergism, additive effect, and antagonism, respectively, whereas DRI = 1, DRI > 1, and DRI < 1 indicate no dose-reduction, favorable dose-reduction, and not favorable dose-reduction, respectively, for each drug in the combination. As shown in Table 2, the combination of lower concentrations of BZ and CFZ (5 + 5 and 10 + 10 nM) showed a moderate synergistic effect and favorable dose reduction for both BZ and CFZ. However, the combination of higher drug concentrations (20 + 20 nM) demonstrated an antagonistic effect and no dose reduction for BZ. Furthermore, the simulated data also demonstrated that the combination of drugs at higher doses, that is, with lower cell growth inhibition effect (Fa: 0.05 and 0.10) could exert antagonistic effect and no or not favorable dose reduction for BZ. These results indicated that the combination of BZ and CFZ in vitro exerted a synergistic effect when the Fa values ranged from 0.15 to 0.95 and the CI values ranged from 0.12 to 0.74.

The synergistic effect of BZ and CFZ in vitro was also confirmed by calculating the expected additive effects (% viability reduction) of combinations of the same concentrations of BZ and CFZ and comparing them with the experimentally derived effects, as suggested by Tallarida [68]. The CFZ-equivalent dose of BZ is given by the expression: b_eq_(a) = Cb/[Eb/Ea(1 + Ca/a)–1], where Ca and Cb are the constants for the doses that give the individual half maximum effect of BZ and CFZ, respectively, and Ea and Eb are the maximum effects for each drug. The modified dose of CFZ (b) is simply the sum of b_o_ and b_eq_(a). Using the modified dose of CFZ, the combination effect on % viability reduction was calculated as follows: E = Eb*b/(b + Cb). Because the effect of the experimental concentrations on cell viability reduction for these combinations was higher than the expected additive effects (62.4% vs. 44.3% at 5 + 5 nM and 85.3% vs. 78.7% at 10 + 10 nM), the effects of BZ and CFZ seemed to be synergistic. However, at the higher concentration, the synergistic or additive effects were not observed, as judged by the lower experimental effect compared to the expected additive effects (87.8% vs. 131.4% at 20 + 20 nM).

Next, the synergistic effect of BZ and CFZ was examined by measuring the levels of cleaved caspases and ER stress-associated proteins. The concentrations of the drugs were set at 25 + 25 nM and 50 + 50 nM at a constant ratio of 1:1. Figure 6B and Figure S9 shows the enhanced activation of caspase 3, GRP78, ATF4, and XBP1 in the 25 + 25 nM combination. However, the combination of drugs at a higher concentration (50 + 50 nM) did not have any effect on the levels of various BZ-induced apoptotic markers, and the levels of some ER stress markers, such as GRP78 and CHOP, decreased. These findings suggested that CFZ at lower concentrations enhanced BZ-induced apoptosis in B16-F1 cells. Taken together, the combination of BZ and CFZ at submaximal concentrations could be a potential treatment strategy for patients with melanoma.

### 2.5. BZ and CFZ Synergistically Reduce Tumor Growth in a Xenograft Model Mouse C57BL/6

**Figure 7:**
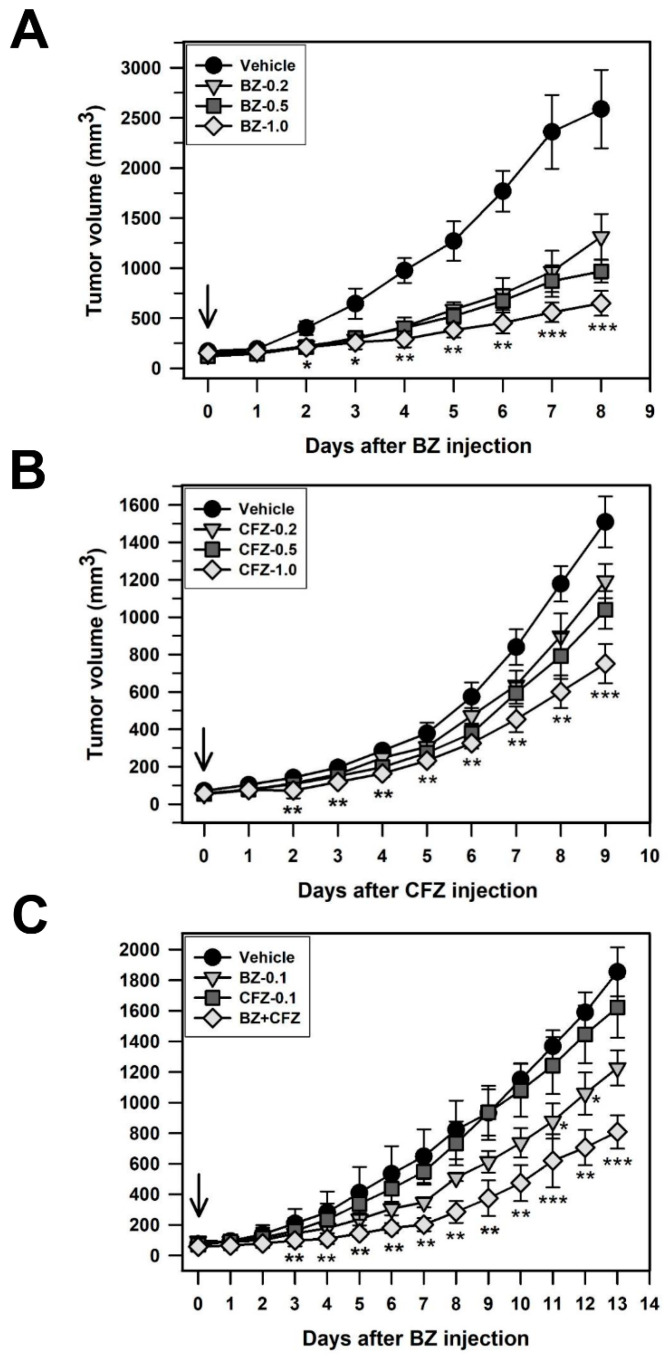
Antitumor effect of BZ and CFZ in C57BL/6 mice. B16-F1 tumor-bearing mice were intraperitoneally injected with vehicle or 0.2, 0.5, and 1.0 mg/kg BZ (**A**) or CFZ (**B**) alone, or a combination of BZ and CFZ at 0.1 mg/kg each (**C**) every 2 days. The tumor volume was recorded every day during the indicated times. For convenience, the means and standard deviations are plotted (*n* = 5, 5, and 6 for biological replicates at each time points in (**A**–**C**), respectively). The Kruskal–Wallis H test followed by Bonferroni-corrected Mann–Whitney U test was used as a post hoc comparison of the daily tumor volume in the syngeneic mouse model. * *p* < 0.05, ** *p* < 0.01, *** *p* < 0.001 compared with the vehicle-injected control mice.

To evaluate the antitumor effect of BZ and CFZ in vivo, a xenograft mouse model was established by injecting B16-F1 cells subcutaneously on the right side of the back of C57BL/6 mice. When the tumor size was approximately 50–100 mm^3^, mice were intraperitoneally injected with BZ, CFZ, or a combination of BZ and CFZ every other day. Tumor volume was measured every day and a significant difference in tumor volumes on each date between the vehicle- and BZ/CFZ-treated groups was identified using the Kruskal–Wallis H nonparametric tests followed by Bonferroni-corrected Mann–Whitney U test (Table 3, Table 4 and Table 5). The percent reductions in tumor growth in the xenograft model mice treated with 0.2, 0.5, and 1.0 mg/kg of BZ were 49.3%, 62.6%, and 74.9% on the final day (8 days), respectively (Figure 7A and Table 3), and those with CFZ were 20.9%, 31.2%, and 50.2% at 9 days, respectively (Figure 7B and Table 4). The degree of tumor growth reduction showed that the effect of BZ was stronger than that of CFZ, as observed in vitro. Combination therapy with 0.1 mg/kg BZ and CFZ was applied to investigate their synergistic effect on the xenograft model mice. As shown in Figure 7C and Table 5, the combination therapy with CFZ and BZ significantly reduced the tumor size compared to that by treatment with either of the drugs alone.

To evaluate the additive and synergistic effects, we calculated the effect of the experimental concentrations on the percent reduction of tumor growth and compared it with the expected additive effect, as suggested by Tallarida [68]. The effect of the experimental concentrations was higher than the expected additive effect at 0.1 + 0.1 mg/kg (56.4% vs. 37.6%); thus, the effects of BZ and CFZ seemed to be synergistic. Therefore, the in vivo experiments also suggested that supplementation of BZ with CFZ might be beneficial in reducing tumor growth and cytotoxicity in patients with melanoma.

## 3. Discussion

Since human melanomas are the most aggressive form of malignant skin cancer, the potential use of BZ in the treatment of metastatic melanoma has been suggested [38]. Indeed, the inhibitory effect of BZ on cell growth has been demonstrated in various malignant human melanoma cell lines, such as Hs 294T, G361, and A375 [57,69,70] and the established human melanoma cell lines from metastatic melanoma lesions [71]. In the present study, we showed the inhibitory effect of BZ on B16-F1 murine melanoma cell growth that has not been reported previously. Furthermore, the effect of epoxomicin, the microbial origin of CFZ, on cell proliferation and apoptosis has been examined in human cutaneous melanoma-derived cell lines [72]. Because the effect of CFZ itself on melanoma cell lines has not been reported previously, we have investigated the effect of CFZ on the cell viability of B16-F1 cells as well as other melanoma cell lines including B16-BL6 with higher metastatic potential; K-1735 carcinogen-induced murine melanoma cell line; and the DX3, A375P (lowly metastatic), and A375SM (highly metastatic) human melanoma cell lines. The data showed that both BZ and CFZ induced morphological changes and cell viability reduction in these melanoma cells (Figure 1).

Human melanomas are the most aggressive form of malignant skin cancer and can be categorized by distinct mutational profiles that determine their corresponding cellular phenotypes, proliferative capabilities, and therapeutic options [8,73]. Sequencing of 108 genes previously implicated in melanogenesis was performed on 462 patient-derived xenografts (PDX), cell lines, and tumors to identify mutational and copy number aberrations [9]. Among them, oncogenic somatic mutations in the *B-Raf* gene (i.e., V599E within the kinase domain) were found in approximately 60% of primary sporadic human melanomas, which result in constitutive activation of the Ser/Thr protein kinase [74]. Oncogenic *B-Raf* leads to hyperactivation of the MEK and Erk/MAPK signaling pathways in a Ras-independent manner. Among the human melanoma cell lines, A375P and A375SM contain the constitutively active oncogenic *B-Raf^V600E^*, whereas the mutational status of *B-Raf* in the human melanoma cell line DX3 is not known, with the p53 gene being intact [75] and MEK appearing to be overexpressed [76].

An analysis of genetic alterations in murine melanoma cell lines demonstrated that unlike human melanomas, B16-F1 and K-1735 do not have activating mutations in the *B-Raf* oncogene [10]. Instead, the expression of p16^Ink4a^ and p19^Arf^ tumor suppressor proteins was lost because of a large deletion spanning the *Ink4a/Arf* exons in the spontaneous B16-F1 melanoma cell line. In contrast, the carcinogen-induced melanoma cell line K-1735 expressed p16Ink4a but had inactivating mutations in the p53 activator *p19^Arf^*. Inactivation of *p19^Arf^* in carcinogen-induced melanomas is accompanied by constitutive activation of MAPKs and/or mutation-associated activation of N-ras. Inactivation of the *Ink4a/Arf* melanoma susceptibility locus has been identified in approximately 20–30% of familial melanoma cases and 15–30% of sporadic melanomas [10,77,78]. Therefore, genetic alterations in the *p16^Ink4a^/p19^Arf^* and ras-MAPK pathways may contribute to the development of murine melanoma. Although B-Raf and MEK inhibitors are actively used for the treatment of metastatic melanoma in patients with *B-Raf^V600E^* mutations, the development of resistance to these drugs has limited their therapeutic utilization in such patients. Moreover, some melanomas expressing wild type B-Raf have intrinsic resistance to B-Raf inhibitors. Since the known therapeutic agents for melanoma treatment have various side effects, the development of safer and more potent treatment strategy is needed.

In the present study, we found that both BZ and CFZ induced cell viability reduction in various melanoma cells, including mouse and human cell lines, regardless of the mutational status of B-Raf (Figure 1A,B, and Table 1). Interestingly, CFZ was less cytotoxic than BZ in all melanoma cells tested in the present study. It is not known why CFZ has a lower cytotoxicity and higher IC_50_ value compared to BZ. Previously, it has been shown that BZ and CFZ have an equal potency to inhibit proteasome activities [79]. However, the other report suggested that CFZ in comparison to BZ exhibits greater selectivity for the chymotrypsin-like activity of the 20S proteasome, accompanied by a more cytotoxic effect than BZ in hematologic tumor cells, but solid tumor cells and non-transformed cell types are less sensitive to a brief CFZ exposure [43]. Because melanoma is also a type of solid tumors, the lower sensitivity to CFZ, compared to BZ, might be a causative factor for its lower cytotoxicity. Otherwise, as suggested by Park et al. [52], the poor aqueous solubility and in vivo instability might be responsible for the reduced dose effect of CFZ in melanoma cells. Even though the application of BZ and CFZ has been considered in many cancers, the efficacy and action mechanisms of these drugs in melanoma have not yet been clarified.

BZ was previously reported to inhibit the growth of various human cancer cell lines in vitro by inhibiting nuclear factor κB (NFκB) activity via the reduction of a proteasomal degradation of inhibitor of NFκB [58,69,70]. However, we did not observe cell cycle arrest by either BZ or CFZ in B16-F1 melanoma cells in the present experimental conditions (Figure 2A). In contrast, BZ and CFZ differentially induced apoptotic cell death, as judged by the increased DNA fragmentation (Figure 2A) and PI/annexin V-FITC double labeled cells (Figure 2B), and caspase activation (Figure 2D–F). Therefore, differential apoptotic cell death might be one of the mechanisms involved in BZ- and CFZ-induced anticancer effect in melanoma cells.

Previously, BZ and CFZ have been shown to increase the cleavage and activation of apoptotic marker caspases 3, 8, and 9 in various leukemia and lymphoma cells [80]. BZ has been shown to trigger apoptosis via activation of capase 9 and 3 in human melanoma cells [57]. CFZ was shown to induce apoptosis via activation of caspases 8, 9, 4, and 3 in BE(2)-M17 human neuroblastoma cells [53]. In addition to caspases 8, 9, and 3, our data showed that BZ and CFZ increased the level of the cleaved caspase 12 (Figure 2D–F), indicating ER stress-associated apoptosis induction in B16-F1 cells. As expected, both BZ and CFZ induced phosphorylation of eIF2α (Figure 3C) and the expression/activation of ER stress-associated proteins, including GRP94, GRP78, ATF6a, ATF4, XBP1, and CHOP, with different concentration- and time-dependencies (Figure 3A,B, respectively). The expression levels of these ER stress-associated proteins correlated well with the induction of apoptosis in BZ- and CFZ-treated cells. Our study also confirmed that ROS accumulation and MMP loss are implicated in BZ- and CFZ-induced ER stress and apoptosis (Figure 4A,B). Interestingly, CHOP expression (Figure 3A,B) and caspase 12 activation (Figure 2F) were decreased at the later time point (24 h) with a greater reduction in CFZ-treated cells compared to BZ-treated cells. Durational differences in CHOP expression and caspase 12 activation between BZ and CFZ might explain their differences in terms of cytotoxicity in B16-F1 cells. However, it is not known how CHOP is rapidly degraded in CFZ-treated cells at this moment.

Combinational drug treatments may provide some benefits as therapeutic strategies for cancer treatment [81]. Since a combination of nivolumab (Opdivo) and ipilimumab (Yervoy), which are monoclonal antibodies and immune checkpoint inhibitors, enhanced T-cell function greater than that with either of the antibodies alone, this combinational therapy was approved for patients with either Stage 3 or Stage 4 melanoma. However, in a phase 3 clinical trial, the 3-year overall survival rate for patients treated with nivolumab alone was 52%, but the combination therapy (nivolumab + ipilimumab) slightly increased the overall survival rate to 58% with serious toxicity [12]. Therefore, a more promising treatment strategy is required for melanoma treatment. In recent years, many researchers have developed new therapeutic approaches that target signaling pathways as well as the immune system. Since B-Raf targeting sensitizes resistant melanoma to cytotoxic T cells, a combination of B-Raf inhibitor and adoptive T-cell therapy could be used in melanomas resistant to B-Raf inhibitors [82].

Previously, researchers have suggested the possibility of synergistic chemosensitization by combining BZ with other chemotherapeutic agents [69,83]. The synergistic effect of two proteasome inhibitors on cancer cell apoptosis has also been demonstrated in a recent study on CFZ and curcumin [84]. In the present study, we found that two proteasome inhibitors, BZ and CFZ, induced apoptosis synergistically via slightly different mechanisms (Figure 6A,B). Indeed, BZ and CFZ synergistically delayed tumor growth in the B16-F1 tumor-bearing mouse model (Figure 7C). Therefore, we suggest the combination of BZ and CFZ as a therapeutic strategy to improve their cytotoxicity against melanoma and reduce the side effects of BZ while retaining the cytotoxicity attained by treatment with BZ alone. The combination of BZ and CFZ can potentially provide benefits in a range of melanomas with distinct mutation profiles.

In response to the UV-induced DNA damage, the skin keratinocytes initially produce the melanocyte-stimulating hormone that binds to melanocortin receptor 1 on the melanocytes that secrete melanin [85]. The melanin pigment initially plays a role in protection against the harmful effects of UV radiation, thus preventing further DNA damage [86]. However, intense and intermittent sun exposure (typical of sunburn history) or chronical sun exposure produces a high mutational load, which is associated with a high risk of cutaneous melanoma. Melanin also has other effects on melanoma behavior and therapy [87]; thus, melanogenesis is regulated by multiple agents via pathways activated by receptor-dependent and receptor-independent mechanisms in a complex multidimensional network [85]. As we did not observe any visible alterations in melanin pigmentation after BZ or CFZ treatment during the experimental periods (data not shown), the melanogenesis regulators were not examined in the present study.

Nonetheless, there is a limitation to our study, which is the use of a syngeneic mouse model bearing tumors originating from only a single murine melanoma cell line. Therefore, our findings should be validated using other types of murine melanoma cell lines, such as the YUMM mouse melanoma cell lines, which are syngeneic to C57Bl/6J, have well defined human-relevant driver mutations, and are genomically stable [61]. Moreover, further studies are needed in well-characterized melanoma patient-derived orthotopic xenograft (PDOX) models to be used as pre-clinical models to facilitate effective drug development [9,17].

## 4. Materials and Methods

### 4.1. Materials

Dulbecco’s modified Eagle’s media (DMEM) and RPMI media were purchased from Corning (Corning, NY, USA). Bortezomib (BZ) and carfilzomib (CFZ) were purchased by Biovision (Mountain View, CA, USA). Fetal bovine serum (FBS), antibiotic-antimycotic agents, trypsin-EDTA, 1 M Hepes, 100 mM L-glutamine, and 100 mM sodium pyruvate were purchased from Gibco/BRL Life Technologies, Inc. (Carlsbad, CA, USA). Minimal Essential Media (MEM) with Eagle’s salts, dimethylthiazol-2-yl)-2,5-diphenyltetrazolium bromide (MTT), dimethyl sulfoxide (DMSO), L-buthionine sulfoximine (BSO), N-acetylcysteine (NAC), propidium idodide (PI), DNase free-RNase A, 2′,7′-dichlorodihydrofluorescein diacetate (DCFH-DA), 3,3′-dihexyloxacarbocyanine iodide (DiOC_6_), and mouse anti-β-actin monoclonal antibody (#A5441) were purchased from Sigma-Aldrich (St. Louis, MO, USA). Antibodies specific for cleaved caspase 3 (#9661), 8 (#8592) and 9 (#9509), caspase 12 (#2202), and phospho-elF2α (Ser51; #3597) were purchased from Cell Signaling (Beverly, MA, USA). Antibodies specific for CHOP/GADD 153 (sc−7351), GRP94 (sc−11402), GRP78 (sc−13968), ATF6α (sc−22799), elF2α (sc−11386), and XBP1 (sc−7160) were purchased from Santa Cruz Biotechnology (Santa Cruz, CA, USA). Antibodies specific for ATF4 (ab23760) were purchased from Abcam (Cambridge, CA, USA). Antibodies specific for horseradish peroxidase (HRP)-conjugated anti-rabbit (PI-1000) and anti-mouse secondary antibodies (PI-2000) were purchased from Vector Laboratories (Burlingame, CA, USA). Cytotoxicity detection kit plus was purchased from Roche Molecular Biochemicals (Indianapolis, IN, USA). Annexin V-Fluorescein isothiocyanate (FITC) apoptosis detection kit was purchased from BD Biosciences (San Diego, CA, USA). ECL Western blot reagent and PVDF and nitrocellulose membranes were purchased from GE healthcare (Fairfield, CT, USA). Bradford protein assay dye reagents and 30% acrylamide/bis-acrylamide solution were purchased from BioRad (Hercules, CA, USA).

### 4.2. Cell Culture

B16-F1 (ATCC^®^ CRL6323™) mouse malignant melanoma cells were purchased from American Type Culture Collection (Manassas, VA, USA) and cultured in DMEM media, which were supplemented with 10% heat-inactivated FBS and antibiotic-antimycotic agents. B16-BL6 (KCLB No. 80006) and K-1735 (KCLB No. 80013) mouse melanoma cells, and DX3 (KCLB No. 80012), A375P (KCLB No. 80003), and A375SM (KCLB No. 80004) human melanoma cells were obtained from Korean Cell Bank (Seoul, Korea) and cultured in MEM media supplemented with 20 mM Hepes, L-glutamine, 1 mM sodium pyruvate, 10% FBS, and antibiotic-antimycotic agents as described previously [88]. All cell lines were maintained in a 5% CO_2_ incubator at 37 °C.

### 4.3. MTT Assay

For a cell viability assay with MTT, cells were seeded at a density of 1 × 10^4^ cells/well and grown for 2 days in a 24-well plate at 37 °C and treated with vehicle or various concentrations of BZ or CFZ alone or in combination for 2 days in the culture medium (*n* = 4 for technical replicates, *n* = 3 for biological replicates). Cells were washed once with 1 mL PBS and incubated with 500 μL MTT stock solution (5 mg/mL in PBS) for 1 h at 37 °C in the dark. The medium was removed and 200 μL DMSO was added to dissolve the formazan in each well. The level of formazan salts was measured at 570 nm using a plate reader (VICTOR^3^, PerkinElmer, Turku, Finland).

### 4.4. Lactate Dehydrogenase Release Assay for Cytotoxicity Detection

Lactate dehydrogenase (LDH) release was determined using a cytotoxicity detection kit plus. B16-F1 cells (3 × 10^3^ cells per well) were grown in a 96-well plate for 2 days. Cells were treated with vehicle, BZ, or CFZ for 24 h (*n* = 4 for technical replicates, *n* = 3 for biological replicates). Fifty microliters of the culture medium was transferred to a fresh plate for the LDH assay. LDH released into the supernatant reduces the tetrazolium salt to the formazan, which was quantitated at 490−492 nm using a plate reader.

### 4.5. Flow Cytometry for DNA Content Analysis and Apoptosis Detection

To examine the DNA content, the ethanol fixed cells were stained with PI and analyzed by flow cytometry [53]. Briefly, B16-F1 cells (4 × 10^4^ cells per dish) were grown for 2 days in a 6-well plate and treated with vehicle, BZ, or CFZ for the indicated times (*n* = 3 for technical replicates, *n* = 3 for biological replicates). For DNA content analysis, the collected cells were fixed in 70% ethanol overnight at 4 °C, washed with PBS, and then treated with 0.2 mg/mL DNase-free RNase A and 20 μg/mL PI solution for 30 min at 37 °C. The PI-stained cells were analyzed using a FACSCalibur flow cytometer (Becton Dickinson, San Jose, CA, USA). For apoptosis detection, the collected cells were washed with ice-cold PBS and stained with PI and annexin V-FITC reagent in the dark. The stained cells were analyzed using FACSCalibur flow cytometer with FL1 and FL3 channels.

### 4.6. Western Blot Analysis

B16-F1 cells (5 × 10^5^ cells per dish) were seeded in 10-cm dishes and grown for 2 days at 37 °C and treated with vehicle or various concentrations (5–200 nM) of BZ or CFZ for 4, 8, 16, or 24 h (*n* = 2 for technical replicates, *n* = 3 for biological replicates). Cells were then washed with ice-cold 1× PBS and total cell extracts were prepared by lysis in ice-cold RIPA buffer containing a 0.5% protease and phosphatase inhibitor cocktail. After clarifying lysates by centrifugation at 14,000× *g* for 20 min at 4 °C, the total protein concentration was determined using the Bradford protein assay reagent. Twenty micrograms of cell lysates were analyzed by Western blotting, as previously described [89]. The blots were incubated with appropriate dilutions of primary and secondary antibodies (1:1000 dilutions for primary antibodies purchased from Cell Signaling; 1:200 dilutions for primary antibodies from Santa Cruz Biotechnology; 1:2000 dilution for monoclonal anti-β-actin antibody; 1:5000 dilutions for HRP-conjugated anti-rabbit or anti-mouse secondary antibodies). The immune complexes were visualized by an ECL detection kit. The protein bands were analyzed by Bio-Rad image analyzer (ChemiDoc MP, Hercules, CA, USA). In our experimental conditions, there was no signal in the negative control blot incubated with 1:5000 dilutions of secondary antibody only.

### 4.7. Measurement of Reactive Oxygen Species Production

For detecting intracellular reactive oxygen species (ROS), DCFH-DA is used. DCFH-DA is a cell-permeable fluorogenic probe that is rapidly hydrolyzed to the DCFH carboxylate anion, which is retained within the cell. Two-electron oxidation of DCFH by intracellular ROS results in the formation of a fluorescent product (DCF), whose fluorescent intensity can be monitored by flow cytometry. For this experiment, B16-F1 cells (4 × 10^4^ cells per well) were seeded and grown in a 6-well plate for 2 days. After treatment with either vehicle, BZ, or CFZ (*n* = 3 for technical replicates, *n* = 3 for biological replicates), cells were treated with 25 μM DCFH-DA for 15 min at 37 °C, as described previously [53]. The fluorescence intensity of collected cells was measured using FACSCalibur flow cytometer with excitation and emission wavelengths of 488 and 525 nm, respectively.

### 4.8. Measurement of Mitochondrial Membrane Potential

Mitochondrial membrane potential (MMP) is an important parameter of mitochondrial function and apoptotic cell death. MMP can be detected by cell permeable lipophilic cationic fluorescent dyes, such as DiOC_6_, which accumulate in mitochondria due to their negative membrane potential [90]. For measuring MMP, B16-F1 cells (4 × 10^4^ cells per well) were seeded in a 6-well plate and incubated for 2 days. After treatment of the cells with vehicle, BZ, or CFZ (*n* = 3 for technical replicates, *n* = 3 for biological replicates), cells were incubated with 10 μM DiOC_6_ for 15 min at 37 °C. The fluorescence intensity of collected cells was measured using a FACSCalibur flow cytometer with excitation and emission wavelengths of 482 and 504 nm, respectively.

### 4.9. Xenograft Model Experiment

Male C57BL/6 mice (6−8 weeks old) were purchased from Orient Bio Inc. (Sungnam, Korea). Mice were kept in a 23 °C and 65% humidity-controlled animal chamber with 12 h light/dark cycles. Mice were given free access to commercial rodent chow and water. Animals were acclimatized for 1 week before use. All procedures for animal experiments were reviewed and approved by the animal care committee of the Center of Animal Care and Use at the Gachon University, Korea (GIACUC-R2017023). B16-F1 cells (2 × 10^6^ in 100 μL PBS) were first injected subcutaneously into one side of flanks of each mouse. One week after tumor cell inoculation, the mice were randomly divided into four groups (*n* = 8 per group). One group was intraperitoneally injected with vehicle as a control group once every 2 days until the end of the experiment. The other groups were intraperitoneally injected with various concentrations of BZ or CFZ, or a combination of BZ and CFZ. Weight and tumor growth were monitored and recorded daily (*n* = 5–6 for biological replicates at each time points). The tumor volume was calculated according to the following formula: 1/2 (Length × Width^2^).

### 4.10. Statistical Analysis

For statistical analyses of in vitro studies, one-way analysis of variance (ANOVA) followed by Dunnett’s T3 post hoc test (SPSS, IBM, Seoul, Korea) was performed to assess significant differences between groups. Statistical analyses with Kruskal–Wallis H nonparametric tests (SPSS; IBM, Seoul, Korea) were performed to assess treatment effects on daily tumor growth in the B16-F1-bearing syngeneic mouse model. As a post hoc comparison on each date after drug injection, Bonferroni-corrected Mann–Whitney U test was performed. *p* < 0.05 was considered statistically significant.

## 5. Conclusions

Both BZ and CFZ lead to apoptosis via ER stress as well as ROS accumulation and MMP loss in melanoma cells. BZ and CFZ synergistically reduced B16-F1 tumor growth in vitro and in a C57BL/6 xenograft mouse model. Therefore, a combination therapy with CFZ and BZ may reduce the side effect of BZ and be beneficial for melanoma treatment.

## Figures and Tables

**Table 1:** In vitro cytotoxicity of BZ and CFZ on various melanoma cells.

Test Melanoma Cells	IC_50_ (nM) *	
	BZ	CFZ
B16-F1 (murine)	8.1	23.9
B16-BL6 (murine, higher metastatic)	10.1	43.4
K-1735 (murine, carcinogen-induced)	5.4	26.1
DX3 (human)	7.1	57.6
A375P (human, low metastatic)	10.4	28.2
A375SM (human, highly metastatic)	7.9	51.0

* IC_50_: the half maximal inhibitory concentration.

**Table 2:** Combination of BZ with CFZ against B16-F1 cell growth in MTT assay in vitro.

Drug		Cell Viability (Fa) ^a^	Parameters ^b^			CI ^c^	DRI ^d^		Comments
BZ (nM)	CFZ (nM)		m	Dm	r		BZ	CFZ	
5	-	0.772	–2.029	8.397	–0.979				
10	-	0.334							
20	-	0.169							
-	5	0.937	–2.002	18.91	–0.999				
-	10	0.769							
-	20	0.481							
5	5	0.376	–1.058	5.263	–0.926	0.669	2.16	4.87	Moderate synergism, favorable dose-reduction
10	10	0.147				0.720	2.00	4.55	
20	20	0.122				1.295	1.11	2.53	Antagonism, no dose- reduction for BZ but favorable for CFZ
		Simulation							
		0.05				1.703	0.84	1.94	Antagonism No or not favorable dose-reduction for BZ but favorable for CFZ
		0.10				1.216	1.18	2.70	
		0.25				0.742	1.94	4.40	Slight synergism, favorable dose-reduction
		0.50				0.453	3.19	7.19	Moderate synergism, favorable dose-reduction
		0.75				0.276	5.24	11.7	High synergism, favorable dose-reduction
		0.90				0.168	8.62	19.1	
		0.95				0.120	12.1	26.7	

This table is formed from the output results generated by CompuSyn Report. ^a^. Dose and data for growth inhibition effect (Fa) were obtained from the MTT assay (average value of quadruplicate) and applied to CompuSyn analysis. ^b^. Parameters were derived from the median-effect equation and plot. The m value is slope; Dm is IC_50_ for BZ or CFZ; and r is linear correlation coefficient. ^c^. Combination index (CI) was calculated from the CI equation algorithms using CompuSyn software. CI = 1, CI < 1, and CI > 1 indicate additive effect, synergism, and antagonism, respectively. ^d^. Dose-reduction index (DRI) was calculated from the DRI equation and algorithm using CompuSyn software. DRI = 1, DRI > 1, and DRI < 1 indicate no dose-reduction, favorable dose-reduction, and not favorable dose-reduction, respectively, for each drug in the combination.

**Table 3:** Kruskal–Wallis H test statistics for the antitumor effect of BZ in B16-F1 tumor-bearing mice.

Days	N	Chi-Square (χ^2^)	Degree of Freedom (df)	Significance (*p*)
0	20	6.020	3	0.111
1	20	3.160	3	0.368
2	20	10.768	3	0.013 *
3	20	11.366	3	0.010 *
4	20	14.154	3	0.003 **
5	20	15.274	3	0.002 **
6	20	15.332	3	0.002 **
7	20	15.754	3	0.001 **
8	20	17.583	3	0.001 **

N: the sum of sample sizes for all samples. * *p* < 0.05, ** *p* < 0.01.

**Table 4:** Kruskal–Wallis H test statistics for the antitumor effect of CFZ in B16-F1 tumor-bearing mice.

Days	N	Chi-Square (χ^2^)	Degree of Freedom (df)	Significance (*p*)
0	20	3.909	3	0.271
1	20	6.020	3	0.111
2	20	13.118	3	0.004 **
3	20	13.674	3	0.003 **
4	20	14.842	3	0.002 **
5	20	12.389	3	0.006 **
6	20	16.419	3	0.001 **
7	20	15.642	3	0.001 **
8	20	15.915	3	0.001 **
9	20	17.010	3	0.001 **

N: the sum of sample sizes for all samples. ** *p* < 0.01.

**Table 5:** Kruskal–Wallis H test statistics for the antitumor effect of BZ + CFZ combination in B16-F1 tumor-bearing mice.

Days	N	Chi-Square (χ^2^)	Degree of Freedom (df)	Significance (*p*)
0	24	6.111	3	0.106
1	24	4.419	3	0.220
2	24	6.099	3	0.107
3	24	11.346	3	0.010 *
4	24	13.614	3	0.003 **
5	24	17.394	3	0.001 **
6	24	18.257	3	0.000 ***
7	24	19.641	3	0.000 ***
8	24	19.667	3	0.000 ***
9	24	19.238	3	0.000 ***
10	24	19.297	3	0.000 ***
11	24	19.262	3	0.000 ***
12	24	19.873	3	0.000 ***
13	24	20.193	3	0.000 ***

N: the sum of sample sizes for all samples. * *p* < 0.05, ** *p* < 0.01, *** *p* < 0.001.

## Data Availability

We presented data obtained from routine in vitro and in vivo experiments. The original Western blot data supporting the figures 2D-F, 3A-C, 5A-B, and 6B were presented as the supplementary figures S1–S9, respectively.

## Supplementary Materials

The following are available online at https://www.mdpi.com/2079-7737/10/2/153/s1.
