# Supplementary material for: Carfilzomib in Combination with Bortezomib Enhances Apoptotic Cell Death in B16-F1 Melanoma Cells
Source: Biology (Basel). 2021 Feb 15;10(2):153. doi: 10.3390/biology10020153 (PMC7918982; doi:10.3390/biology10020153)
Supplement: Supplementary file 1 [file biology-10-00153-s001.pdf]

Supplementary Figure Legends

Figure S1. A representative whole blot for western blot analysis of each protein in Figure 2D. Cells were treated with various concentrations of BZ for 24 h in 2% FBS/DMEM or 10% FBS/DMEM. Total cell extracts were analyzed by western blotting with antibodies against the cleaved forms of caspases (Cas-3, -8, -9 and -12) and β-actin. The band densities were normalized against β-actin and the fold changes compared to that of vehicle-treated control (0 nM) are written under each band. A representative whole blot for western blot analysis of each protein are shown below each band.

Figure S2. A representative whole blot for western blot analysis of each protein in Figure 2E. Cells were treated with various concentrations of CFZ for 24 h in 2% FBS/DMEM or 10% FBS/DMEM. Total cell extracts were analyzed by western blotting with antibodies against the cleaved forms of caspases (Cas-3, -8, -9 and -12) and β-actin. The band densities were normalized against β-actin and the fold changes compared to that of vehicle-treated control (0 nM) are written under each band. A representative whole blot for western blot analysis of each protein are shown below each band.

Figure S3. A representative whole blot for western blot analysis of each protein in Figure 2F. Cells were treated with 100 nM BZ or CFZ for the indicated times in 2% FBS/DMEM. Total cell extracts were analyzed by western blotting with antibodies against the cleaved forms of caspases (Cas-3, -8, -9 and -12) and β-actin. The band densities were normalized against β-actin and the fold changes compared to that of vehicle-treated control (–/– at 4 h) are written under each band. A representative whole blot for western blot analysis of each protein are shown below each band.

Figure S4. A representative whole blot for western blot analysis of each protein in Figure 3A. Cells were treated with vehicle or various concentrations of BZ or CFZ for 24 h. Cell lysates were analyzed by western blotting with antibodies against ER stress-associated proteins (GRP94, GRP78, p90 ATF6α, ATF4, XBP1, and CHOP) and β-actin. The band densities were normalized against β-actin and the fold changes compared to that of vehicle-treated control (0 nM) are written under each band. A representative whole blot for western blot analysis of each protein are shown below each band.

Figure S5. A representative whole blot for western blot analysis of each protein in Figure 3B. Cells were treated with vehicle or 100 nM BZ or CFZ for the indicated times in 2% FBS/DMEM. Cell lysates were analyzed by western blotting with antibodies against ER stress-associated proteins (GRP94, GRP78, p90 ATF6α, XBP1, CHOP) and β-actin. The band densities were normalized against β-actin and the fold changes compared to that of vehicle-treated control (-/- at 4 h) are written under each band. A representative whole blot for western blot analysis of each protein are shown below each band.

Figure S6. A representative whole blot for western blot analysis of each protein in Figure 3C. Cells were treated with vehicle or 100 nM BZ or CFZ for the indicated times in 2% FBS/DMEM (B and C). Cell lysates were analyzed by western blotting with antibodies against

ER stress-associated proteins (P-eIF2α, eIF2α, and ATF4) and β-actin. The band densities were normalized against β-actin and the fold changes compared to that of early time point (2 h in C) are written under each band. A representative whole blot for western blot analysis of each protein are shown below each band.

Figure S7. A representative whole blot for western blot analysis of each protein in Figure 5A. Cells were pretreated with 5 mM NAC for 1 h and treated with a vehicle, 100 nM BZ or 100–200 nM CFZ for 24 h in 2% FBS/DMEM. Cell lysates were analyzed by western blotting with antibodies against cleaved caspase-3, CHOP, and β-actin. The band densities were normalized against β-actin and the fold changes compared to that in vehicle-treated control are indicated below each band. A representative whole blot for western blot analysis of each protein are shown below each band.

Figure S8. A representative whole blot for western blot analysis of each protein in Figure 5B. Cells were pretreated with 100 μM BSO (B) for 24 h and treated with a vehicle, 100 nM BZ or 100–200 nM CFZ for 24 h in 2% FBS/DMEM. Cell lysates were analyzed by western blotting with antibodies against cleaved caspase-3, CHOP, and β-actin. The band densities were normalized against β-actin and the fold changes compared to that in vehicle-treated control are indicated below each band. A representative whole blot for western blot analysis of each protein are shown below each band.

Figure S9. A representative whole blot for western blot analysis of each protein in Figure 6B. Cells were treated with BZ or CFZ alone, or combinations of BZ and CFZ for 24 h in 2% FBS/DMEM. Total cell extracts were analyzed by western blotting with antibodies against cleaved caspases, ER stress-associated proteins and β-actin. The band densities were normalized against β-actin and the fold changes compared to that of vehicle-treated control (–/–) are written under each band. A representative whole blot for western blot analysis of each protein are shown below each band.

Supplementary Figures

Figure S1 (suppl. data for Fig. 2D)

S1-1

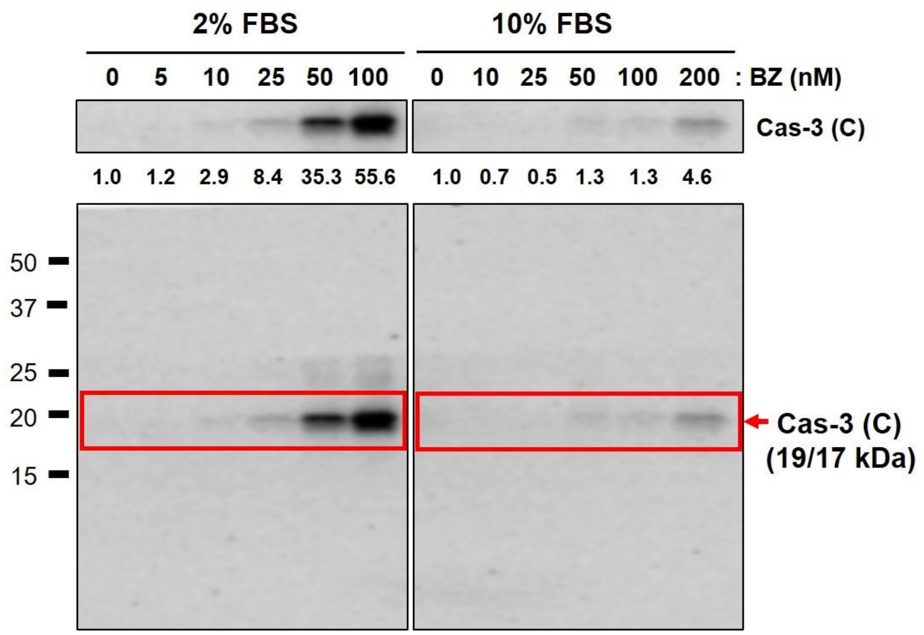

S1-2

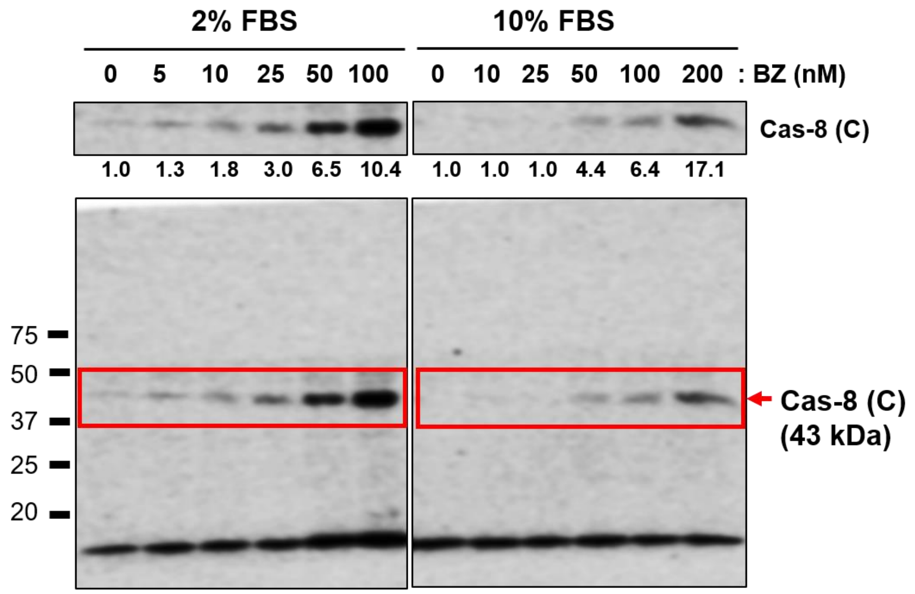

S1-3

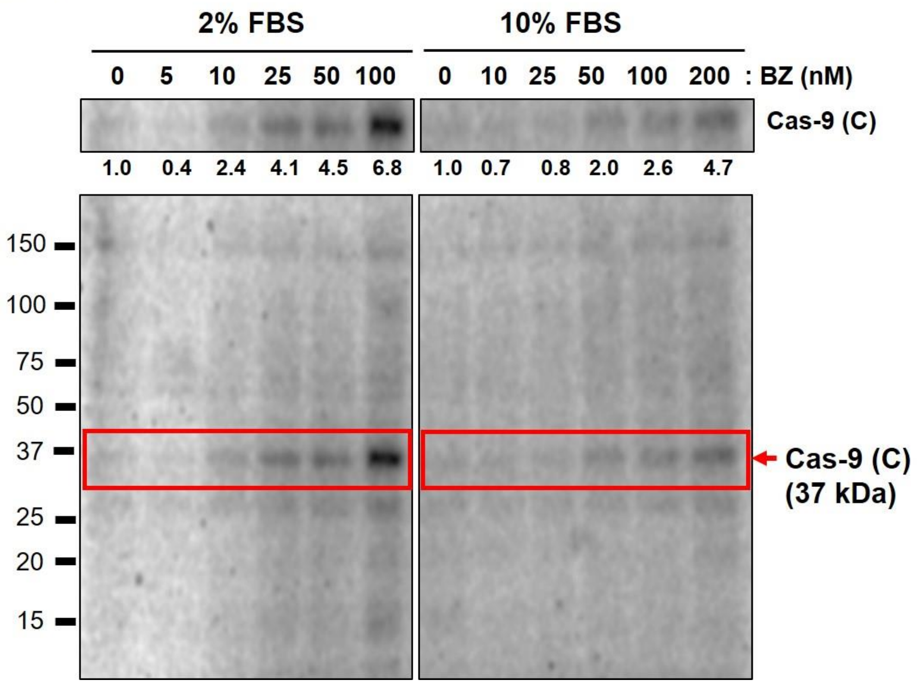

2% FBS
10% FBS
0 5 10 25 50 100
0 10 25 50 100 200 : BZ (nM)
Cas-9 (C)
1.0 0.4 2.4 4.1 4.5 6.8
1.0 0.7 0.8 2.0 2.6 4.7
150
100
75
50
37
25
20
15
Cas-9 (C)
(37 kDa)

S1-4

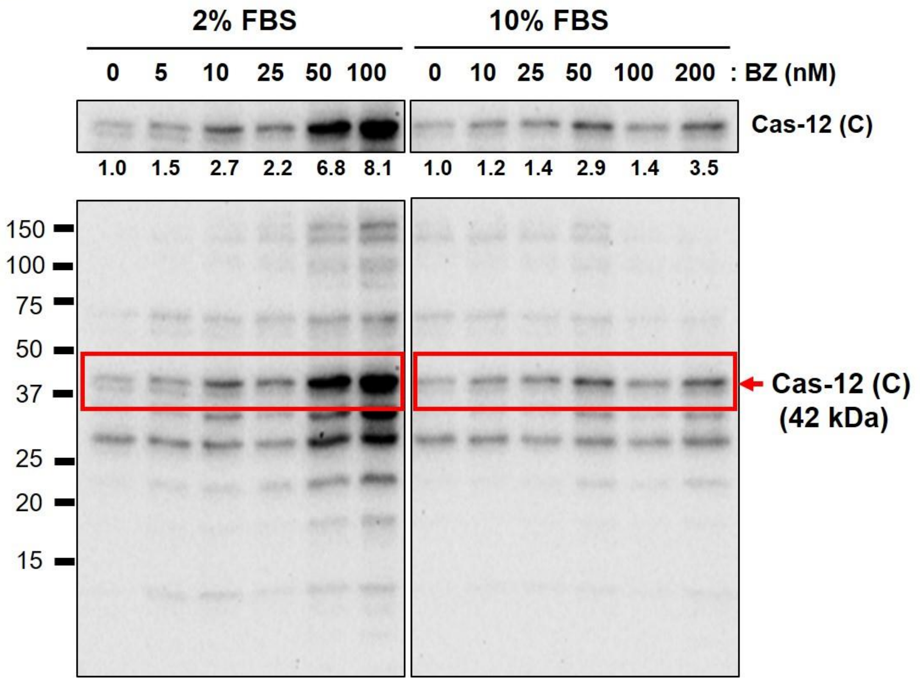

2% FBS
10% FBS
0 5 10 25 50 100
0 10 25 50 100 200 : BZ (nM)
Cas-12 (C)
1.0 1.5 2.7 2.2 6.8 8.1
1.0 1.2 1.4 2.9 1.4 3.5
150
100
75
50
37
25
20
15
Cas-12 (C)
(42 kDa)

S1-5

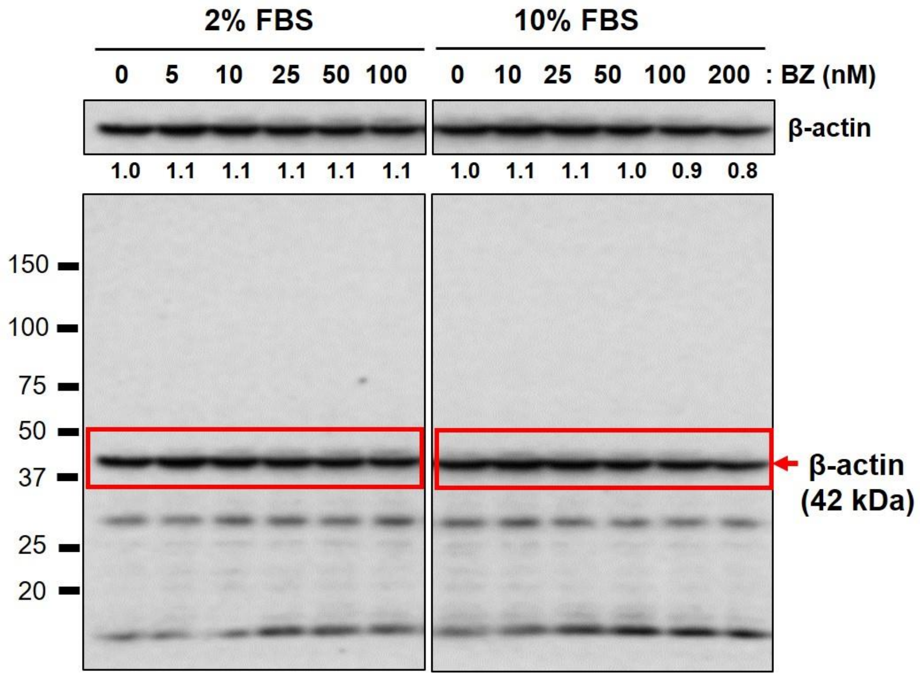
2% FBS
10% FBS
0 5 10 25 50 100
0 10 25 50 100 200 : BZ (nM)
β-actin
1.0 1.1 1.1 1.1 1.1 1.1
1.0 1.1 1.1 1.0 0.9 0.8
150
100
75
50
37
25
20
β-actin
(42 kDa)

Figure S2 (suppl. data for Fig. 2E)

S2-1

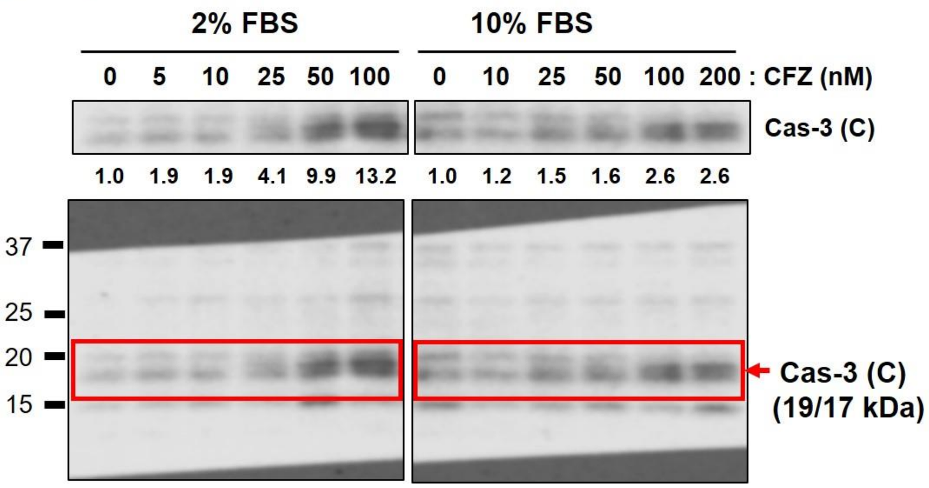

S2-2

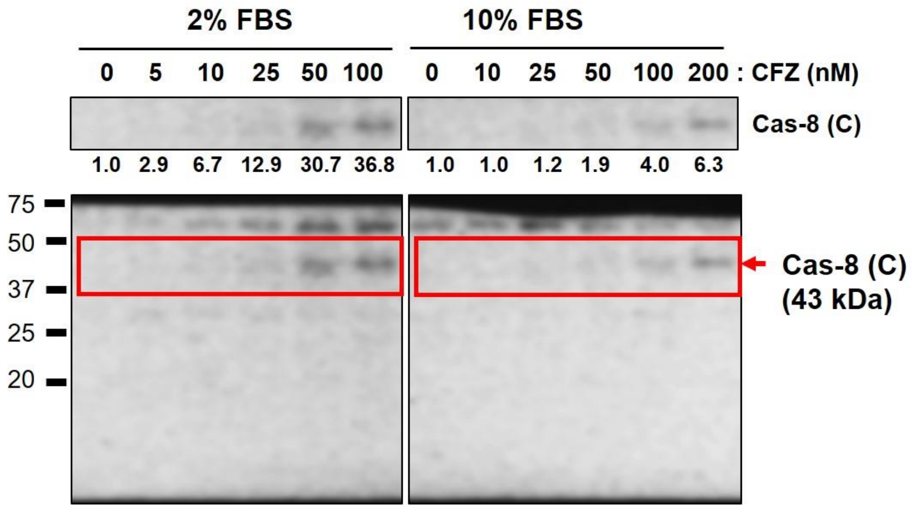

S2-3

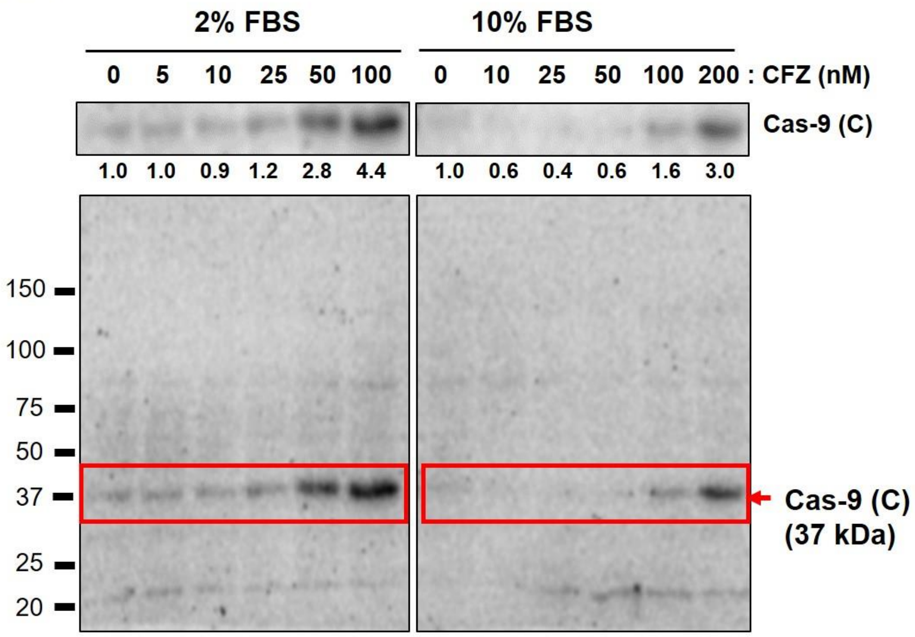

2% FBS
10% FBS
0 5 10 25 50 100
0 10 25 50 100 200 : CFZ (nM)
Cas-9 (C)
1.0 1.0 0.9 1.2 2.8 4.4
1.0 0.6 0.4 0.6 1.6 3.0
150
100
75
50
37
25
20
Cas-9 (C)
(37 kDa)

S2-4

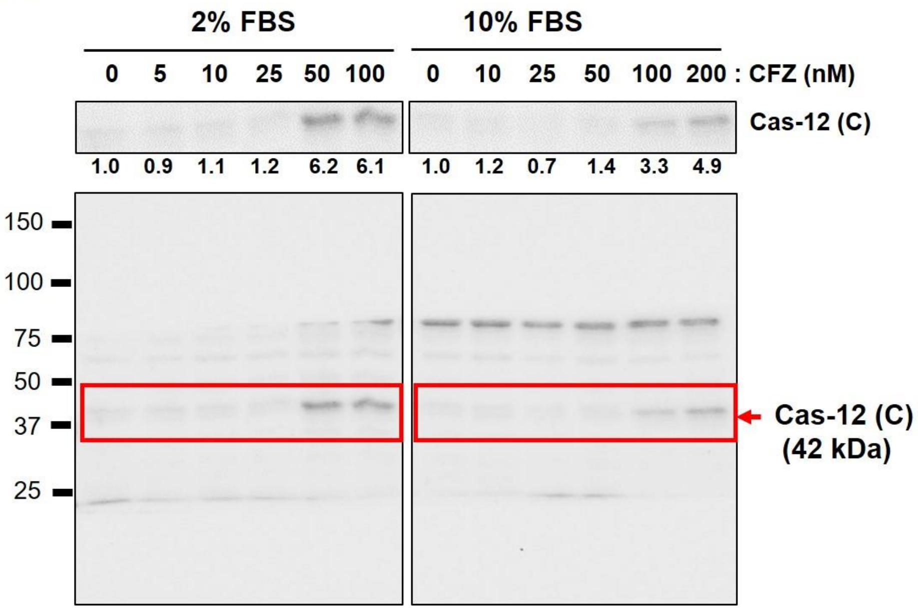

2% FBS
10% FBS
0 5 10 25 50 100
0 10 25 50 100 200 : CFZ (nM)
Cas-12 (C)
1.0 0.9 1.1 1.2 6.2 6.1
1.0 1.2 0.7 1.4 3.3 4.9
150
100
75
50
37
25
Cas-12 (C)
(42 kDa)

S2-5

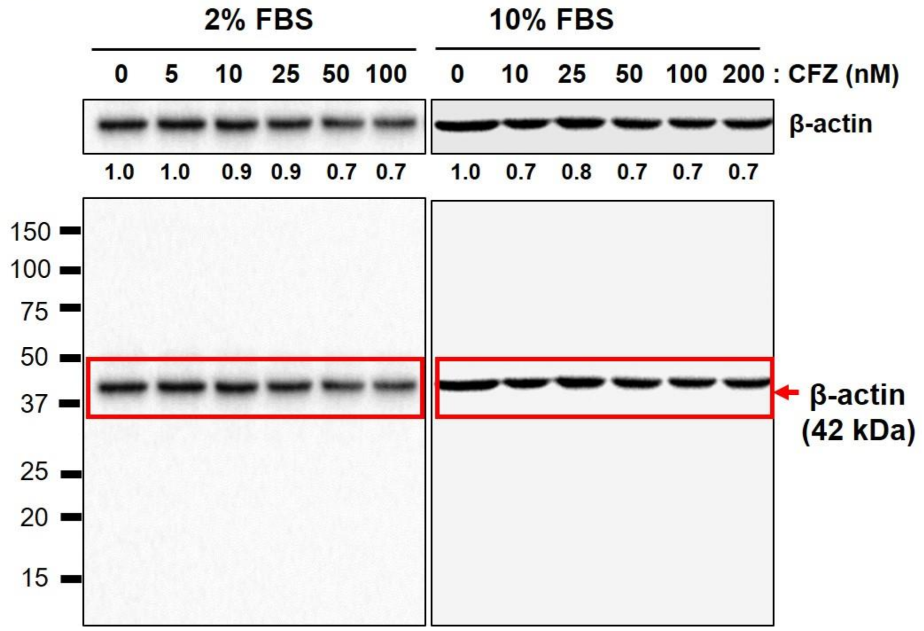
2% FBS
10% FBS
0 5 10 25 50 100
0 10 25 50 100 200 : CFZ (nM)
β-actin
1.0 1.0 0.9 0.9 0.7 0.7
1.0 0.7 0.8 0.7 0.7 0.7
150
100
75
50
37
25
20
15
β-actin
(42 kDa)

Figure S3 (suppl. data for Fig. 2F)

S3-1

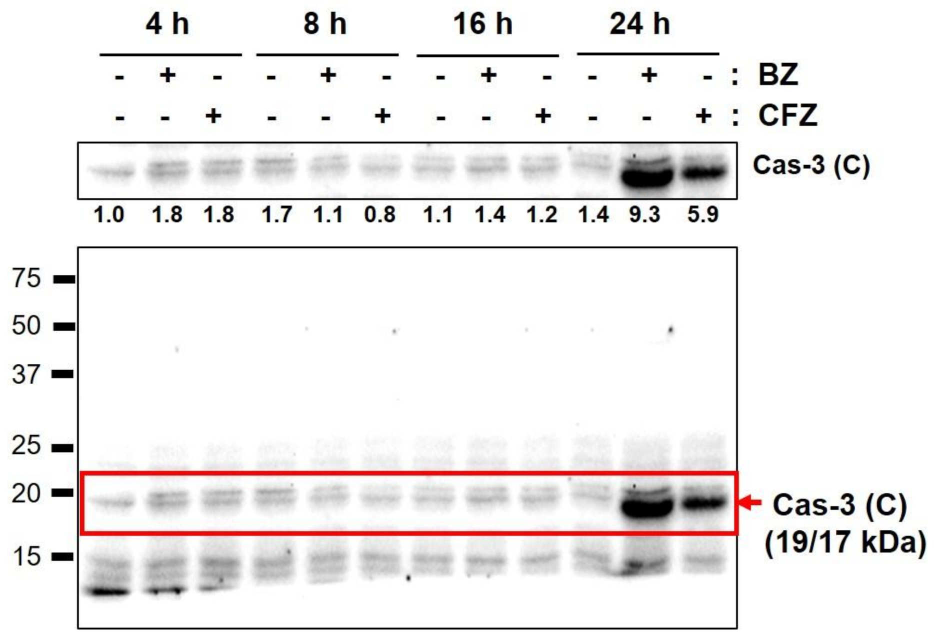

S3-2

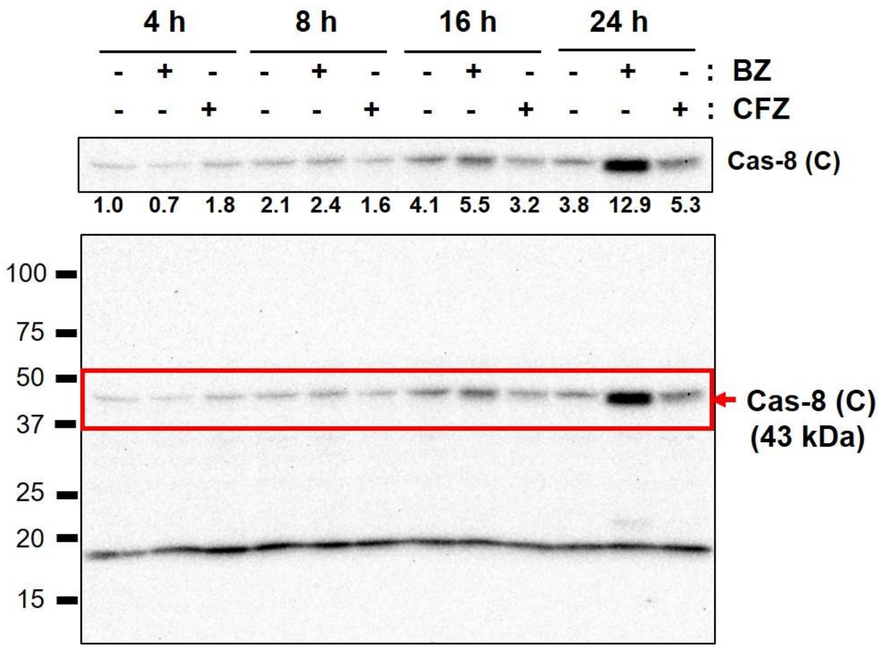

S3-3

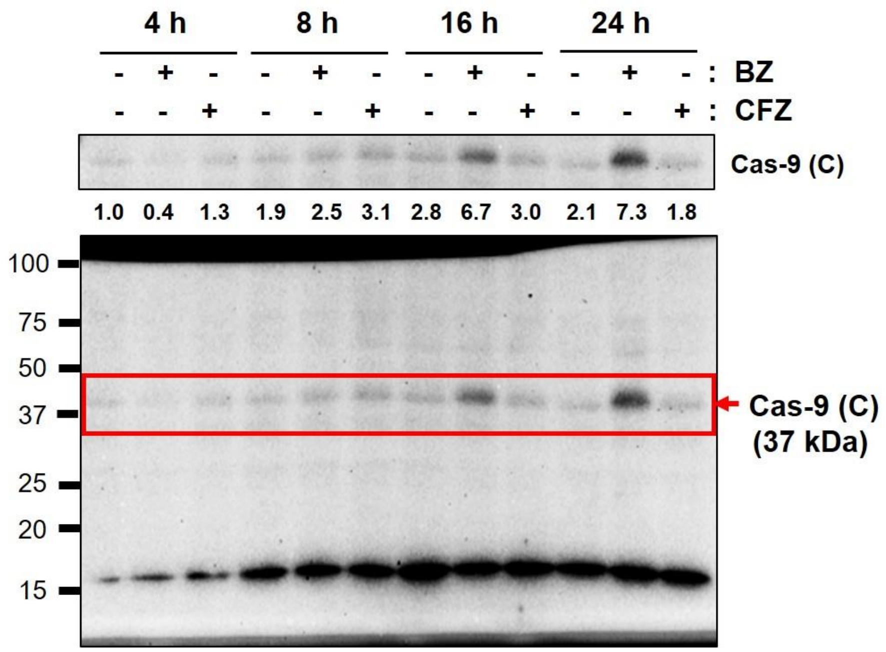
4 h
8 h
16 h
24 h
- + - - + - - + - - + - : BZ
- - + - - + - - + - - + : CFZ
Cas-9 (C)
1.0 0.4 1.3 1.9 2.5 3.1 2.8 6.7 3.0 2.1 7.3 1.8
100
75
50
37
25
20
15
Cas-9 (C)
(37 kDa)

S3-4

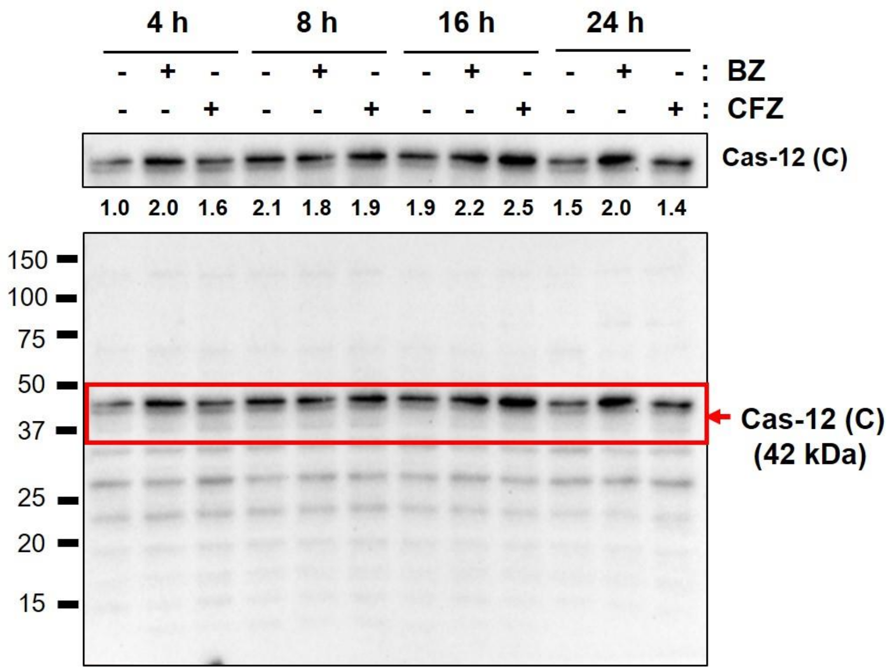
4 h
8 h
16 h
24 h
- + - - + - - + - - + - : BZ
- - + - - + - - + - - + : CFZ
Cas-12 (C)
1.0 2.0 1.6 2.1 1.8 1.9 1.9 2.2 2.5 1.5 2.0 1.4
150
100
75
50
37
25
20
15
Cas-12 (C)
(42 kDa)

S3-5

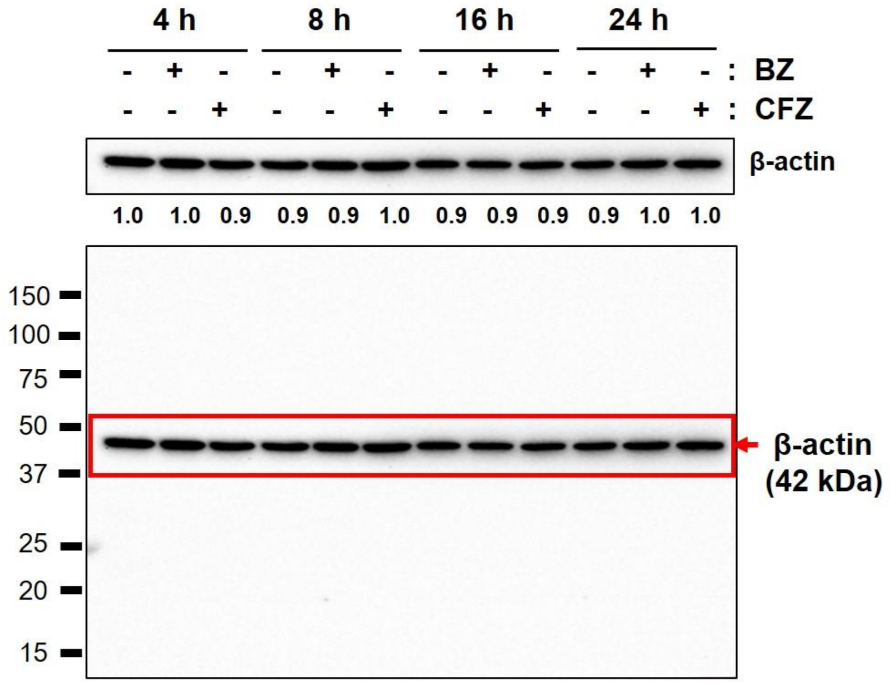

4 h
8 h
16 h
24 h
- + - - + - - + - - + - : BZ
- - + - - + - - + - - + : CFZ
β-actin
1.0 1.0 0.9 0.9 0.9 1.0 0.9 0.9 0.9 0.9 1.0 1.0
150
100
75
50
37
25
20
15
β-actin
(42 kDa)

Figure S4 (suppl. data for Fig. 3A)

S4-1

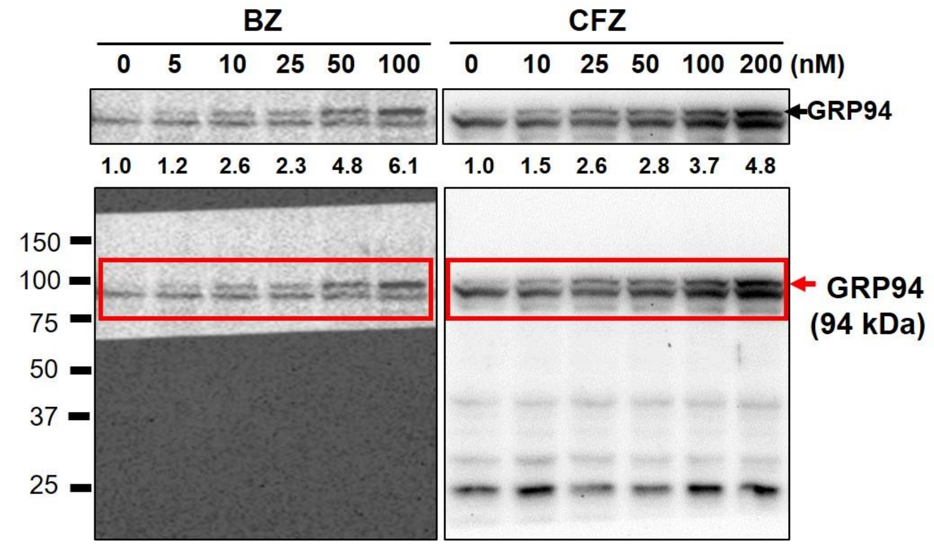

S4-2

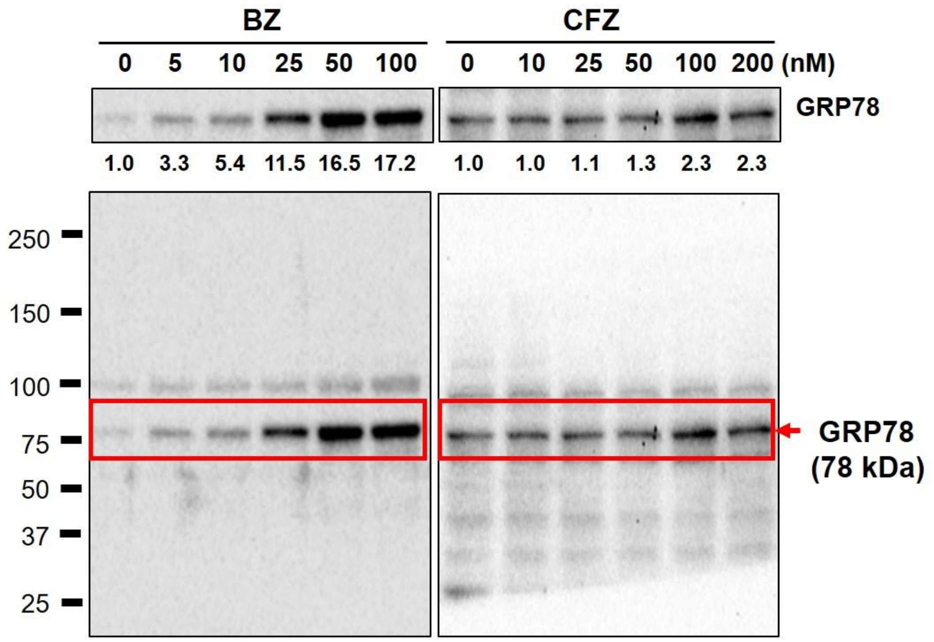

S4-3

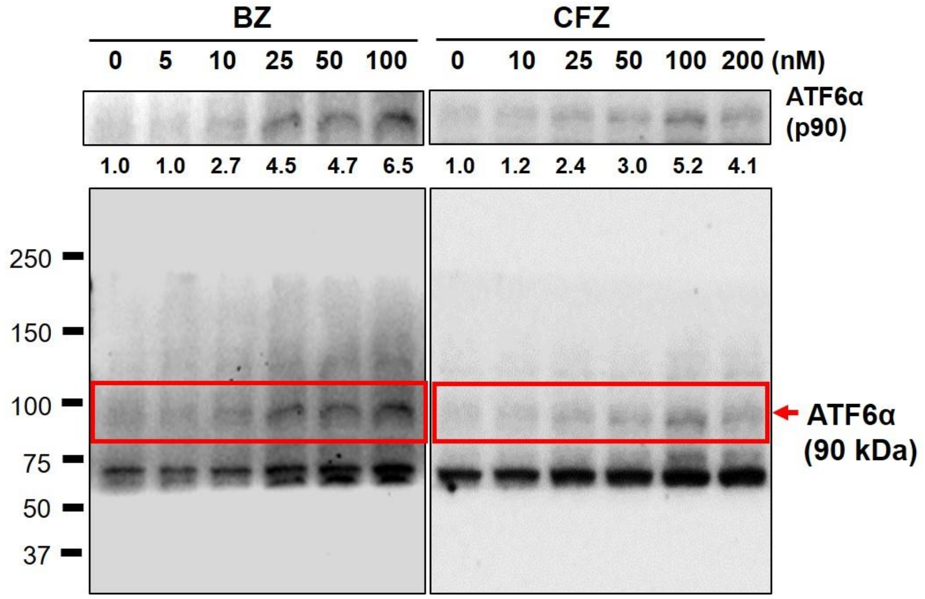
BZ
CFZ
0 5 10 25 50 100
0 10 25 50 100 200 (nM)
ATF6α (p90)
1.0 1.0 2.7 4.5 4.7 6.5
1.0 1.2 2.4 3.0 5.2 4.1
250
150
100
75
50
37
ATF6α (90 kDa)

S4-4

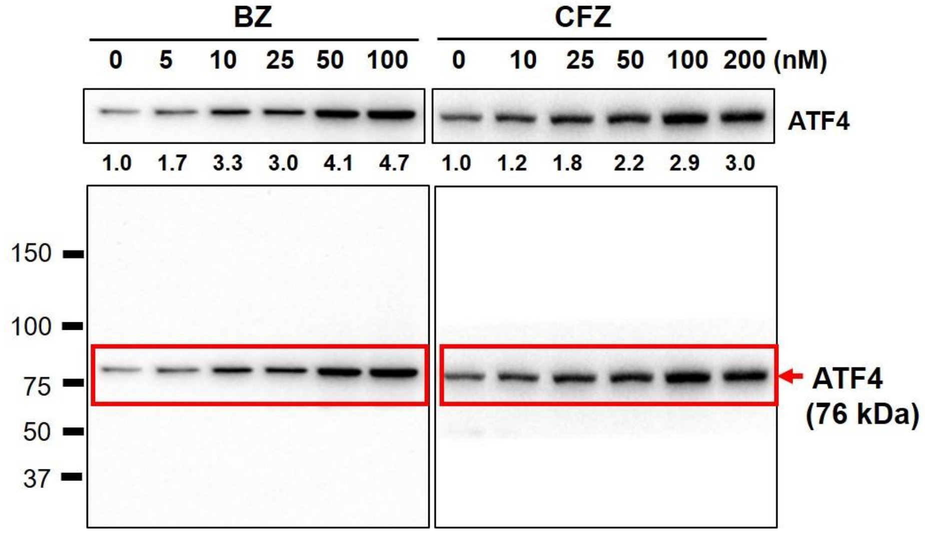
BZ
CFZ
0 5 10 25 50 100
0 10 25 50 100 200 (nM)
ATF4
1.0 1.7 3.3 3.0 4.1 4.7
1.0 1.2 1.8 2.2 2.9 3.0
150
100
75
50
37
ATF4 (76 kDa)

S4-5

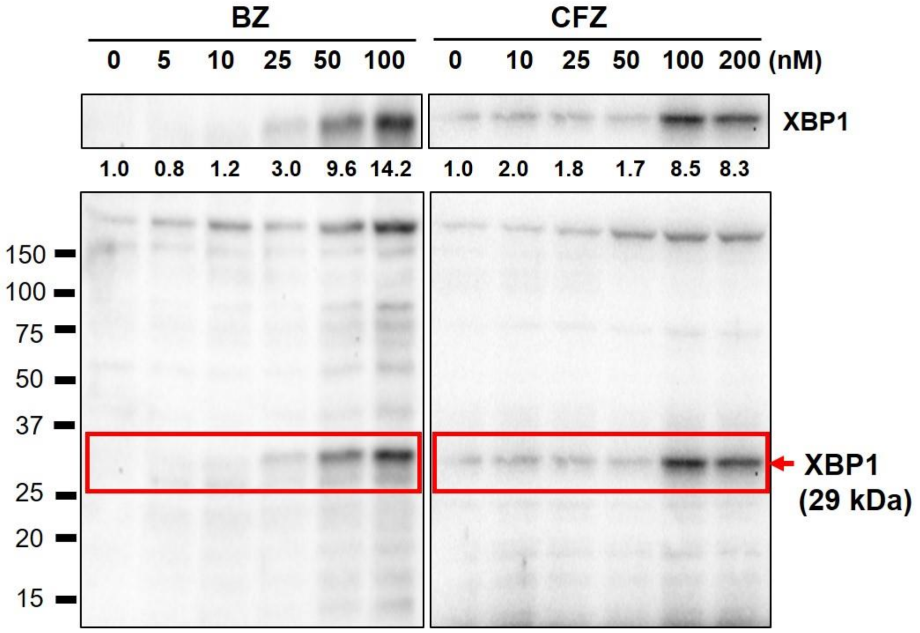
BZ
CFZ
0 5 10 25 50 100
0 10 25 50 100 200 (nM)
XBP1
1.0 0.8 1.2 3.0 9.6 14.2
1.0 2.0 1.8 1.7 8.5 8.3
150
100
75
50
37
25
20
15
XBP1
(29 kDa)

S4-6

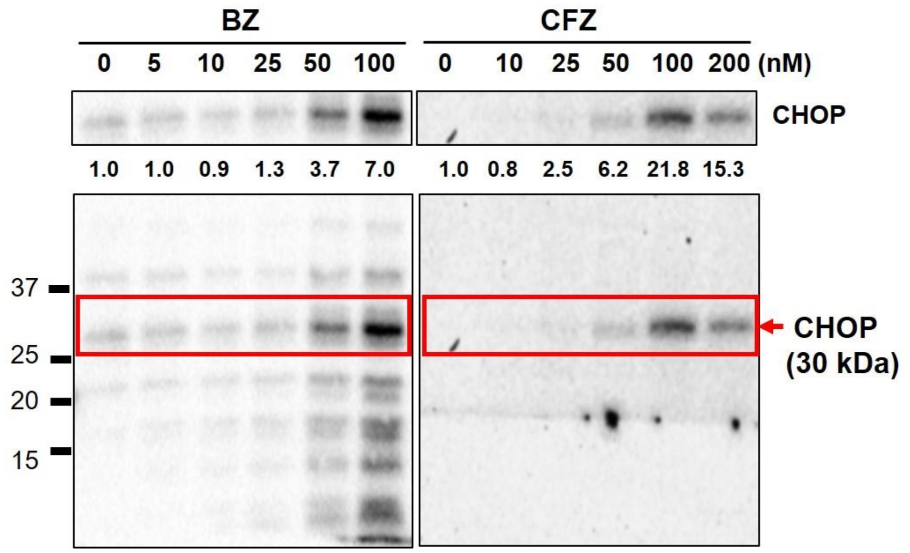
BZ
CFZ
0 5 10 25 50 100
0 10 25 50 100 200 (nM)
CHOP
1.0 1.0 0.9 1.3 3.7 7.0
1.0 0.8 2.5 6.2 21.8 15.3
37
25
20
15
CHOP
(30 kDa)

S4-7

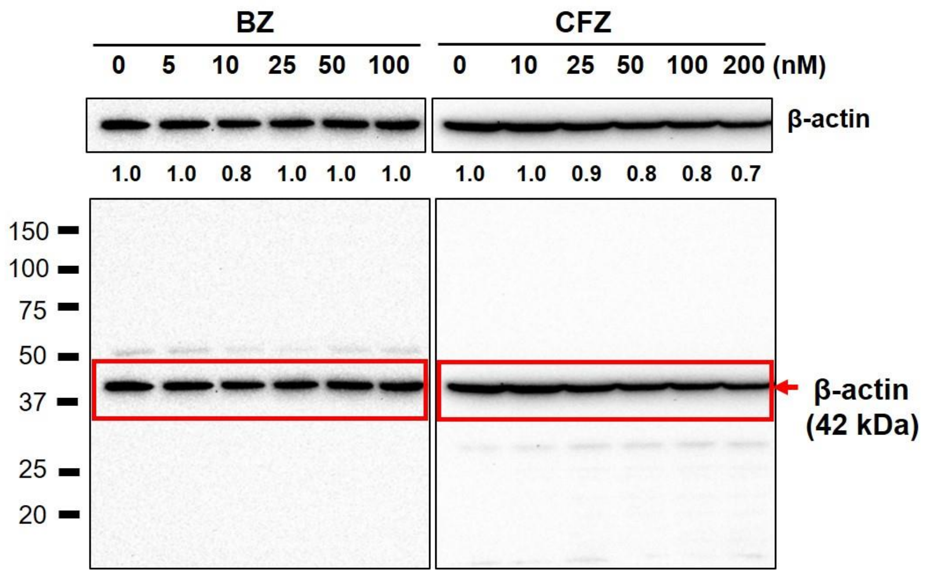
BZ
CFZ
0 5 10 25 50 100
0 10 25 50 100 200 (nM)
β-actin
1.0 1.0 0.8 1.0 1.0 1.0
1.0 1.0 0.9 0.8 0.8 0.7
150
100
75
50
37
25
20
β-actin
(42 kDa)

Figure S5 (suppl. data for Fig. 3B)

S5-1

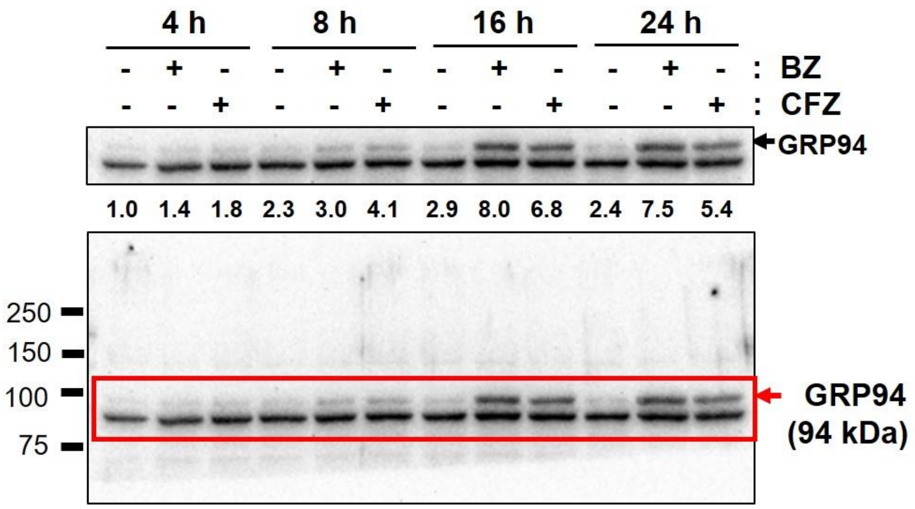

S5-2

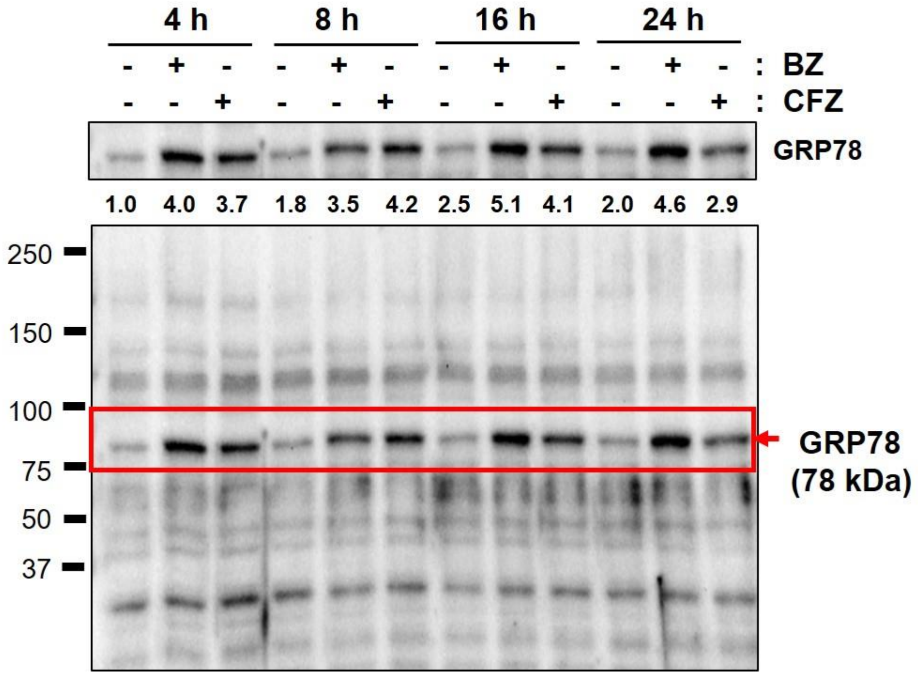

S5-3

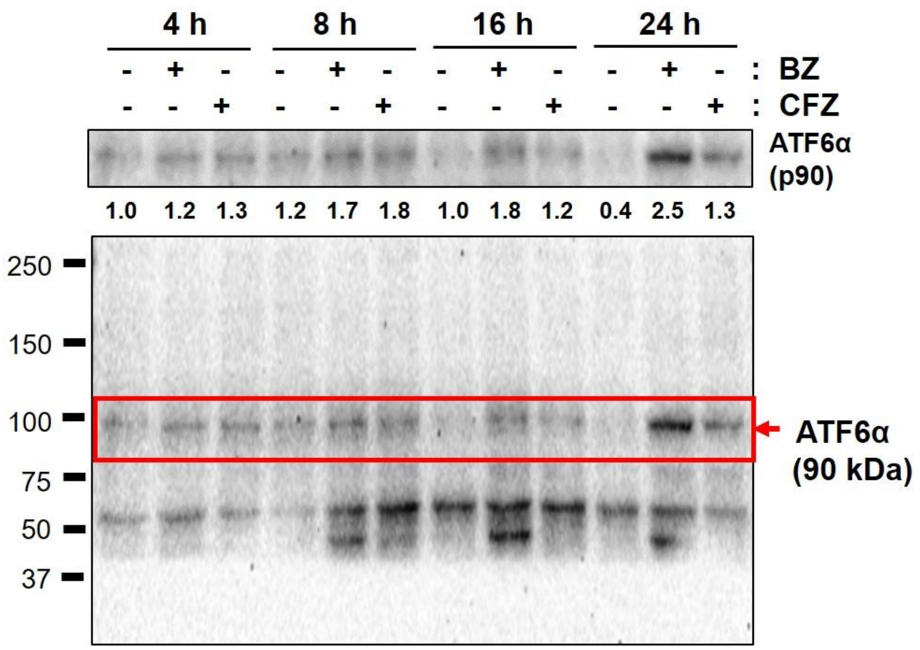
4 h
8 h
16 h
24 h
- + - - + - - + - - + - : BZ
- - + - - + - - + - - + : CFZ
ATF6α
(p90)
1.0 1.2 1.3 1.2 1.7 1.8 1.0 1.8 1.2 0.4 2.5 1.3
250
150
100
75
50
37
ATF6α
(90 kDa)

S5-4

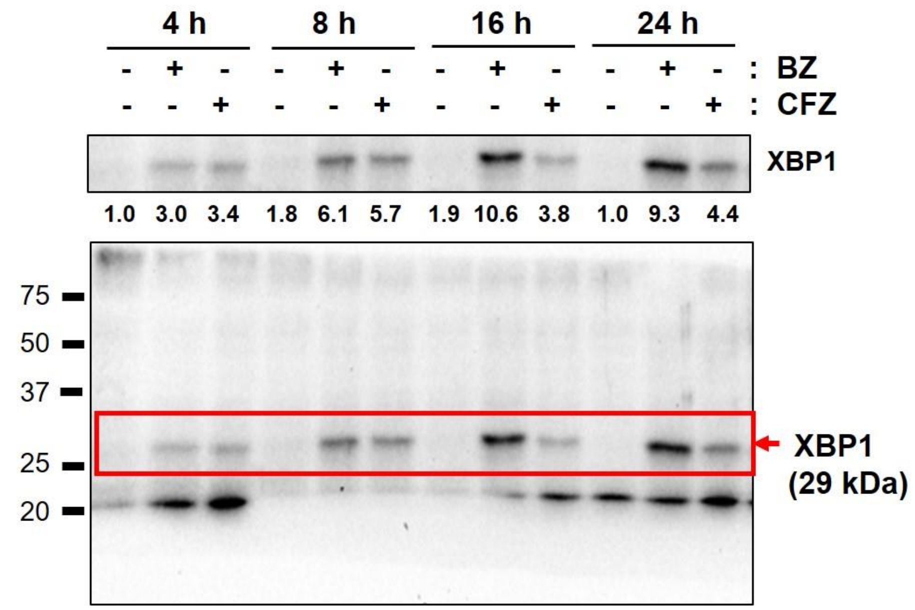
4 h
8 h
16 h
24 h
- + - - + - - + - - + - : BZ
- - + - - + - - + - - + : CFZ
XBP1
1.0 3.0 3.4 1.8 6.1 5.7 1.9 10.6 3.8 1.0 9.3 4.4
75
50
37
25
20
XBP1
(29 kDa)

S5-5

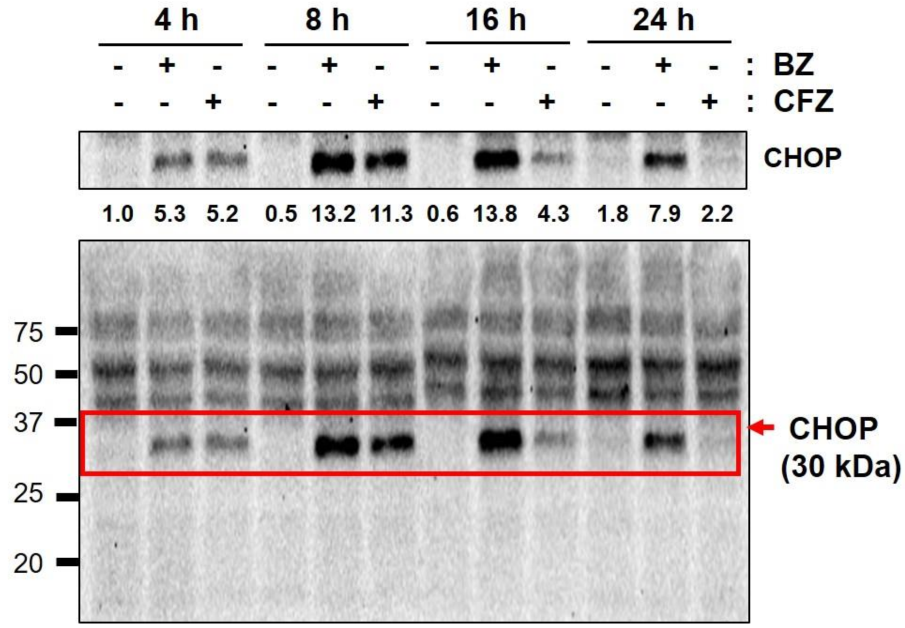
4 h
8 h
16 h
24 h
- + - - + - - + - - + - : BZ
- - + - - + - - + - - + : CFZ
CHOP
1.0 5.3 5.2 0.5 13.2 11.3 0.6 13.8 4.3 1.8 7.9 2.2
75
50
37
25
20
CHOP
(30 kDa)

S5-6

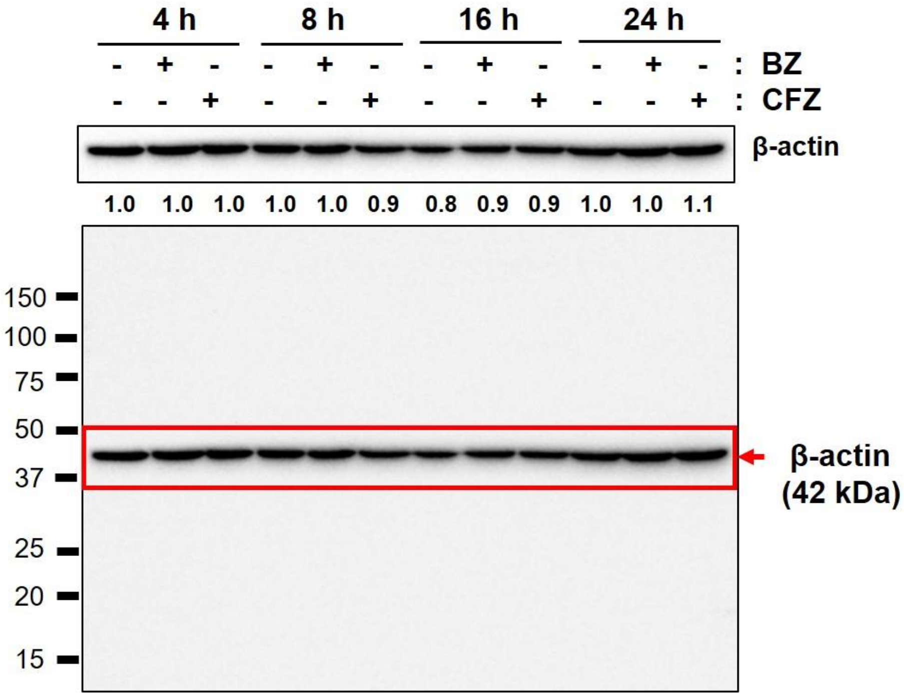
4 h
8 h
16 h
24 h
- + - - + - - + - - + - : BZ
- - + - - + - - + - - + : CFZ
β-actin
1.0 1.0 1.0 1.0 1.0 0.9 0.8 0.9 0.9 1.0 1.0 1.1
150
100
75
50
37
25
20
15
β-actin
(42 kDa)

Figure S6 (suppl. data for Fig. 3C)

S6-1

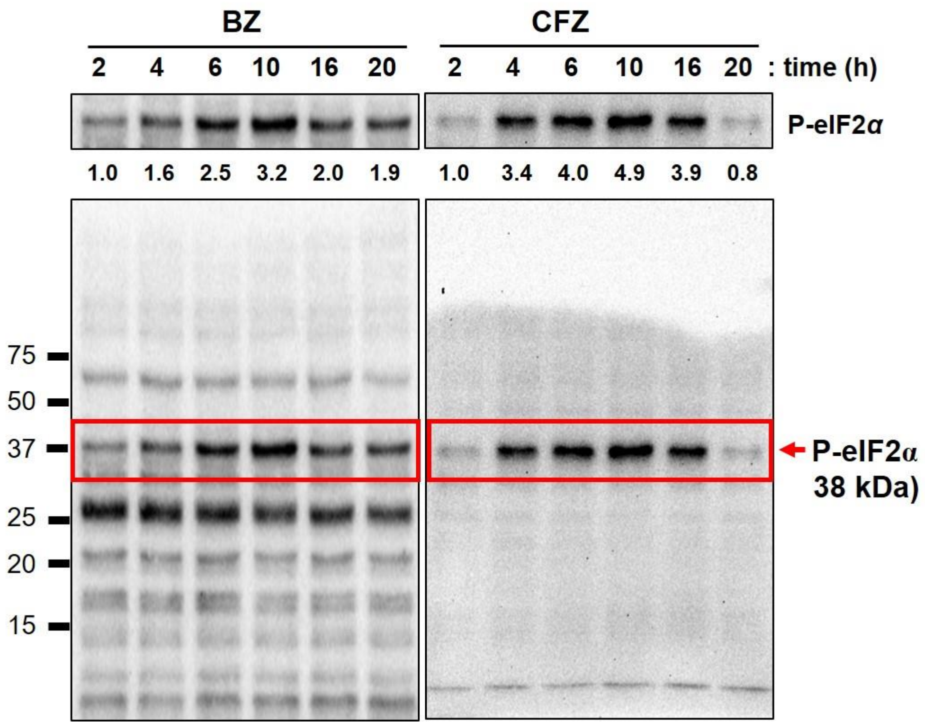

S6-2

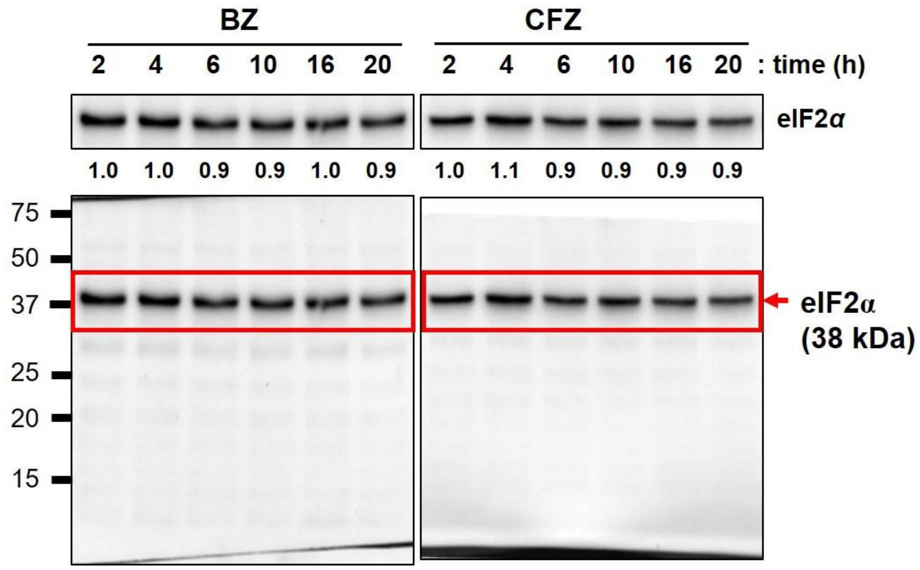

S6-3

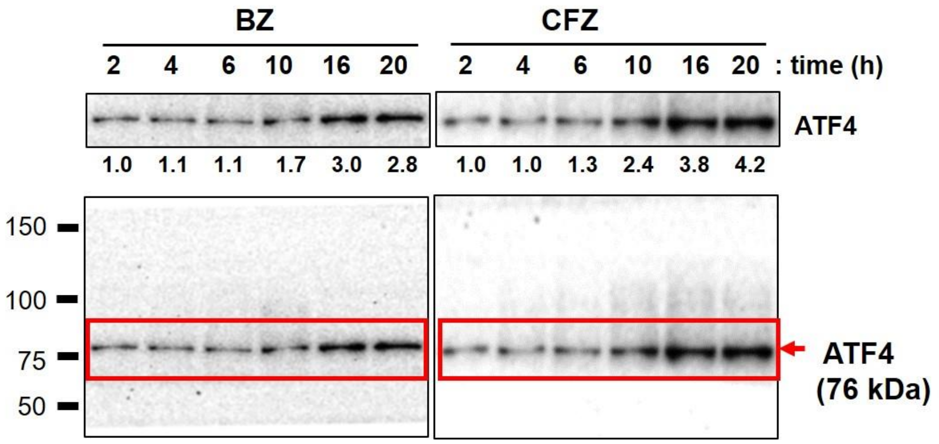

BZ
CFZ
2 4 6 10 16 20 2 4 6 10 16 20 : time (h)
ATF4
1.0 1.1 1.1 1.7 3.0 2.8 1.0 1.0 1.3 2.4 3.8 4.2
150
100
75
50
ATF4
(76 kDa)

S6-4

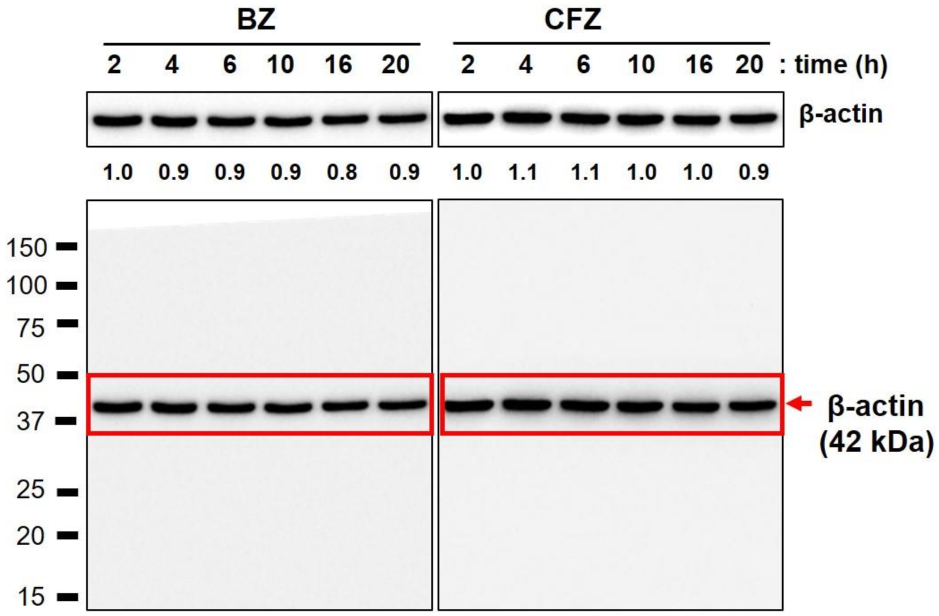

BZ
CFZ
2 4 6 10 16 20 2 4 6 10 16 20 : time (h)
β-actin
1.0 0.9 0.9 0.9 0.8 0.9 1.0 1.1 1.1 1.0 1.0 0.9
150
100
75
50
37
25
20
15
β-actin
(42 kDa)

Figure S7 (suppl. data for Fig. 5A)

S7-1

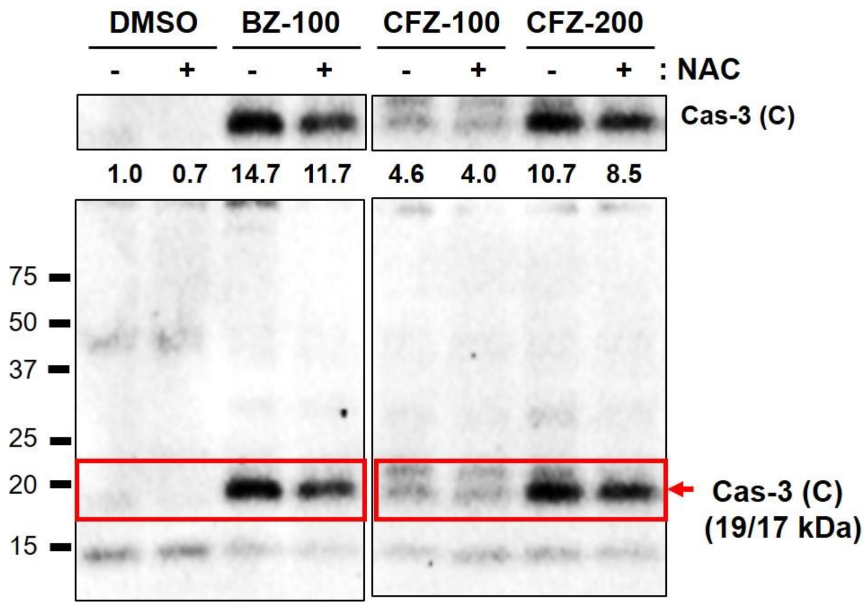

S7-2

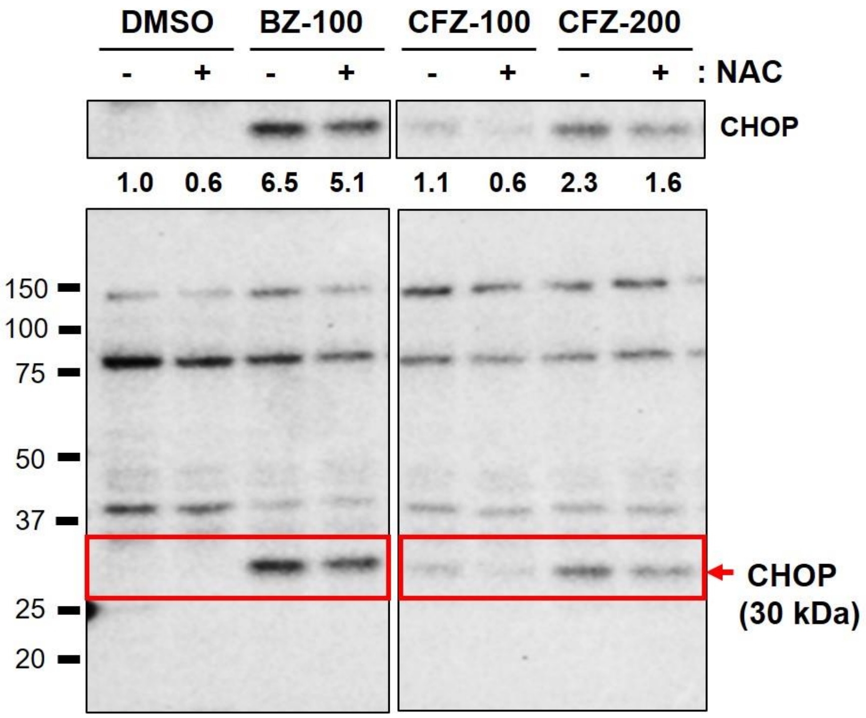

S7-3

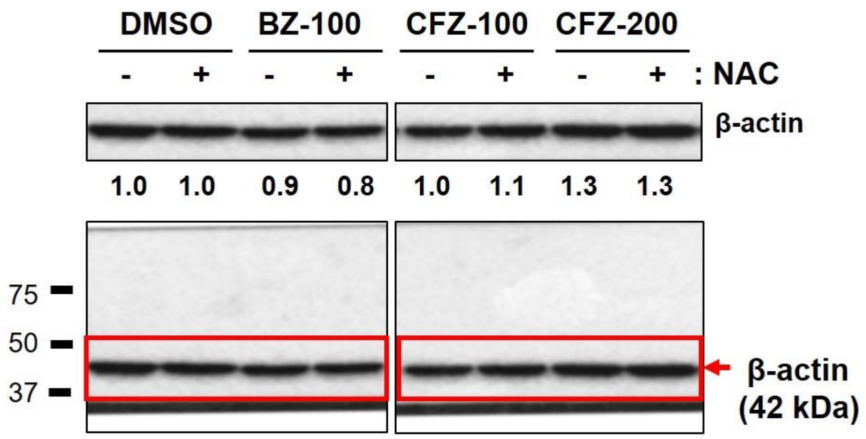

DMSO
BZ-100
CFZ-100
CFZ-200
- + - + - + - + : NAC
β-actin
1.0 1.0 0.9 0.8 1.0 1.1 1.3 1.3
75
50
37
β-actin
(42 kDa)

Figure S8 (suppl. data for Fig. 5B)

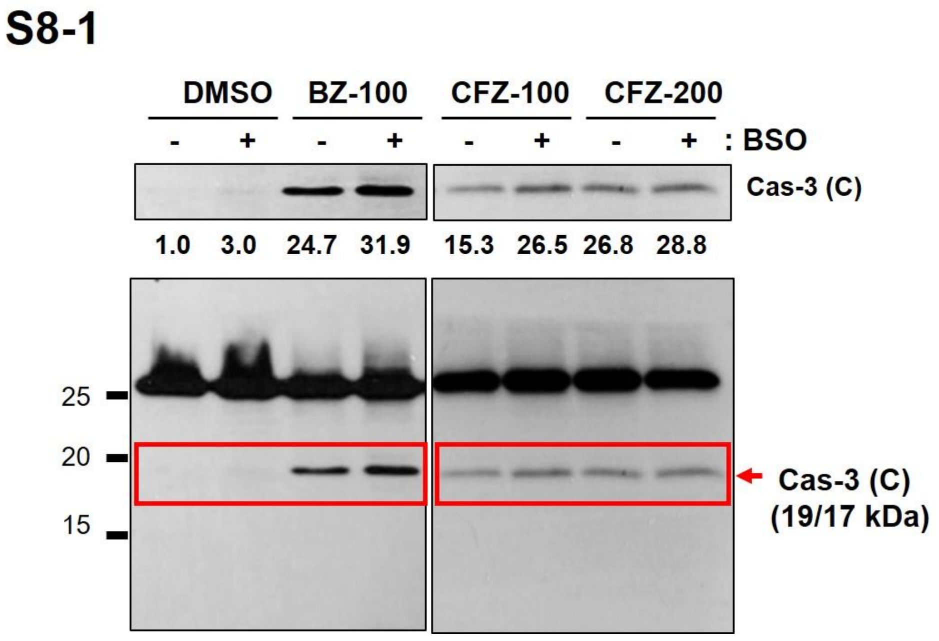

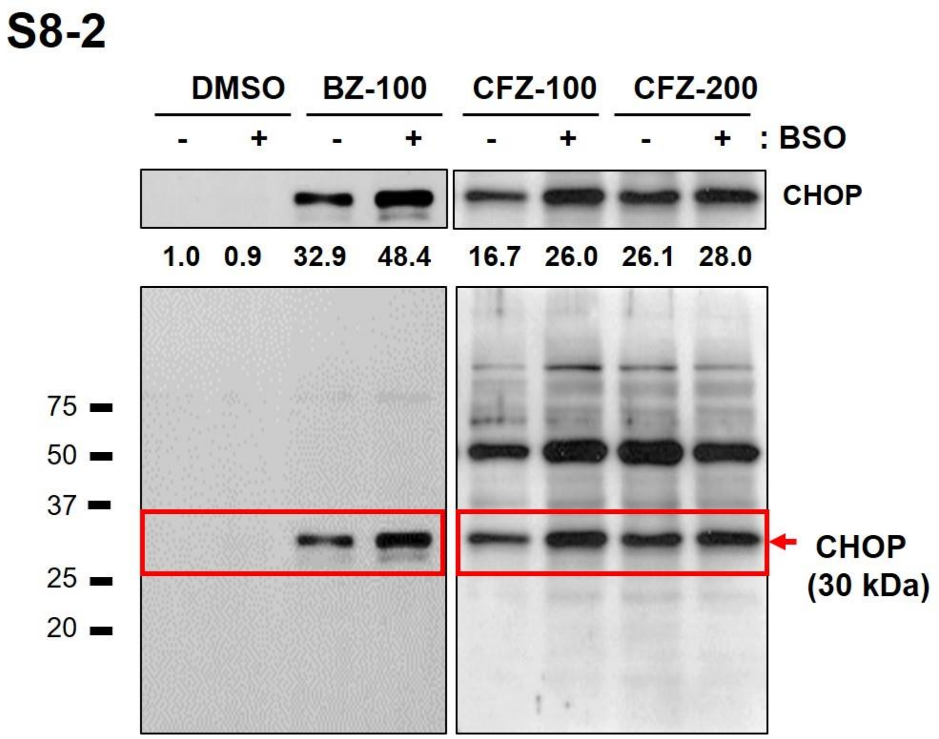

S8-3

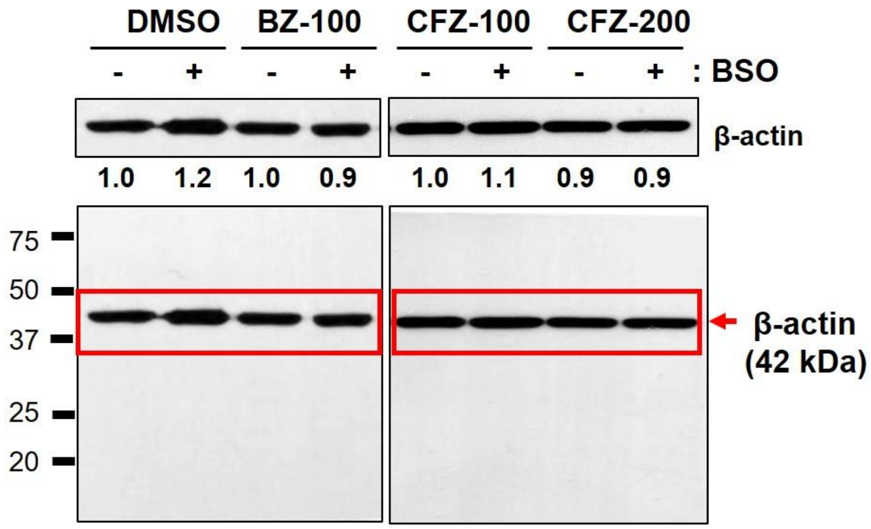
DMSO
BZ-100
CFZ-100
CFZ-200
- + - + - + - + : BSO
β-actin
1.0 1.2 1.0 0.9 1.0 1.1 0.9 0.9
75
50
37
25
20
β-actin
(42 kDa)

Figure S9 (suppl. data for Fig. 6B)

S9-1

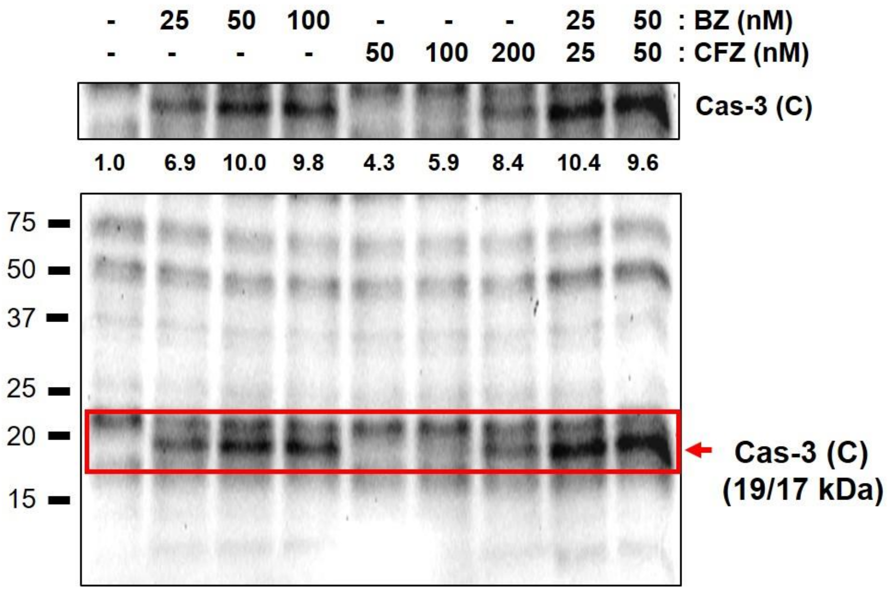

S9-2

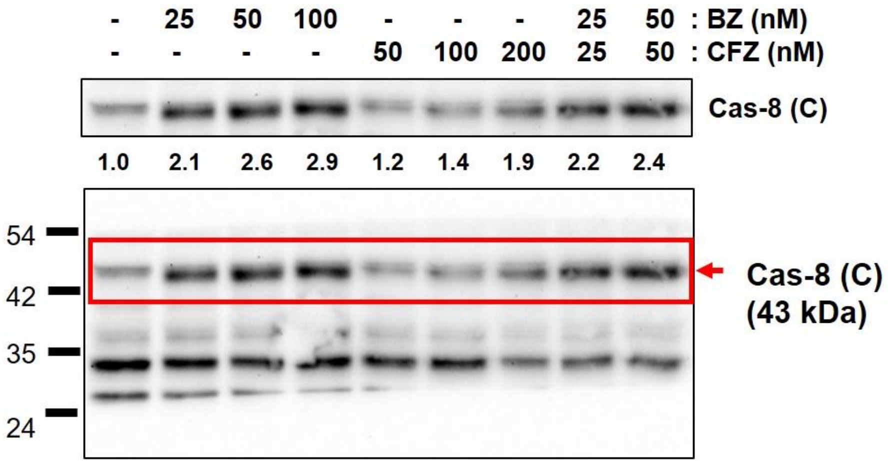

S9-3

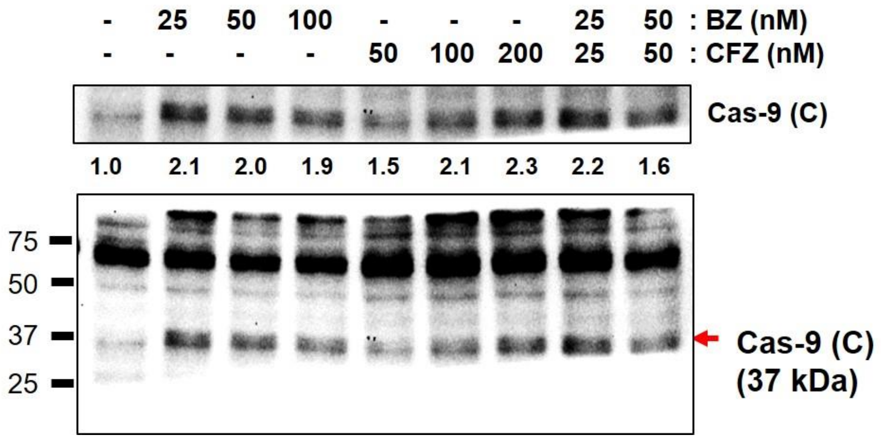
- 25 50 100 - - - 25 50 : BZ (nM)
- - - - 50 100 200 25 50 : CFZ (nM)
Cas-9 (C)
1.0 2.1 2.0 1.9 1.5 2.1 2.3 2.2 1.6
75
50
37
25
Cas-9 (C)
(37 kDa)

S9-4

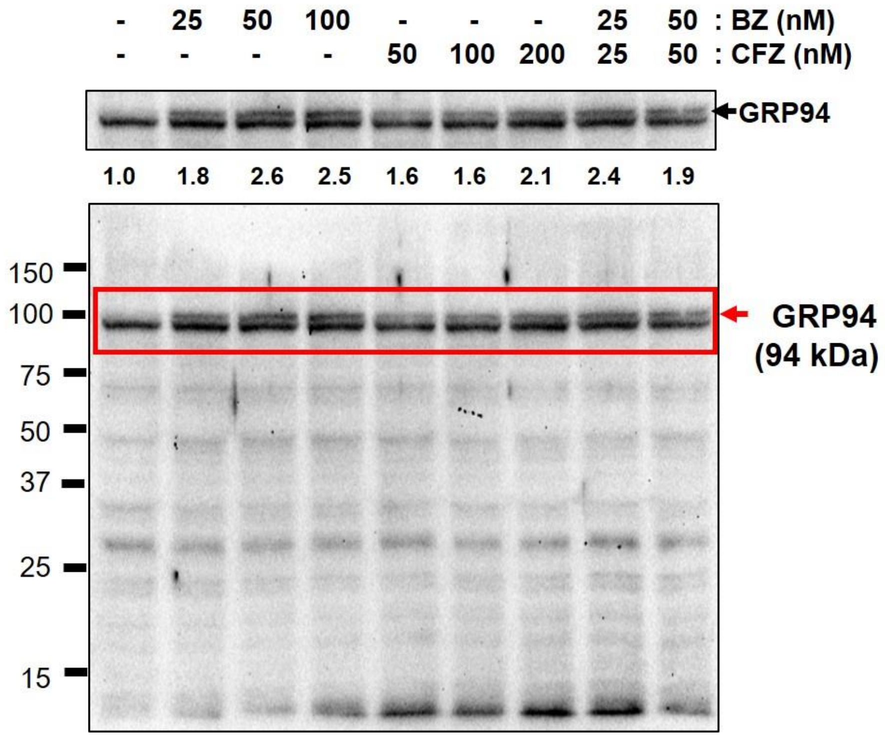
- 25 50 100 - - - 25 50 : BZ (nM)
- - - - 50 100 200 25 50 : CFZ (nM)
GRP94
1.0 1.8 2.6 2.5 1.6 1.6 2.1 2.4 1.9
150
100
75
50
37
25
15
GRP94
(94 kDa)

S9-5

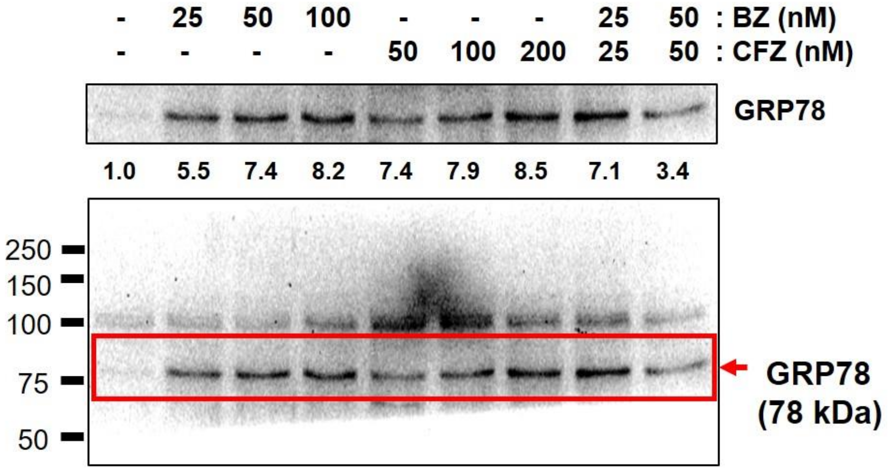

- 25 50 100 - - - 25 50 : BZ (nM)
- - - - 50 100 200 25 50 : CFZ (nM)
GRP78
1.0 5.5 7.4 8.2 7.4 7.9 8.5 7.1 3.4
250
150
100
75
50
GRP78
(78 kDa)

S9-6

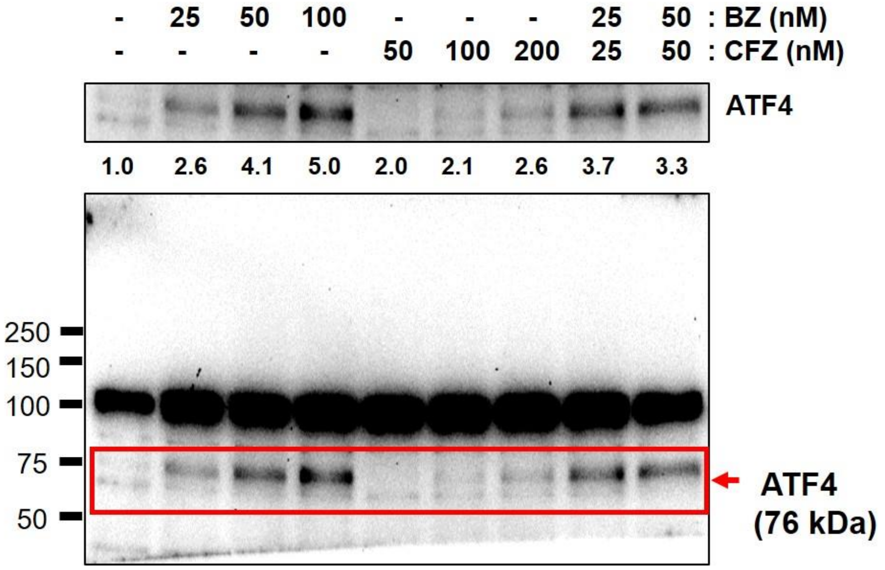

- 25 50 100 - - - 25 50 : BZ (nM)
- - - - 50 100 200 25 50 : CFZ (nM)
ATF4
1.0 2.6 4.1 5.0 2.0 2.1 2.6 3.7 3.3
250
150
100
75
50
ATF4
(76 kDa)

S9-7

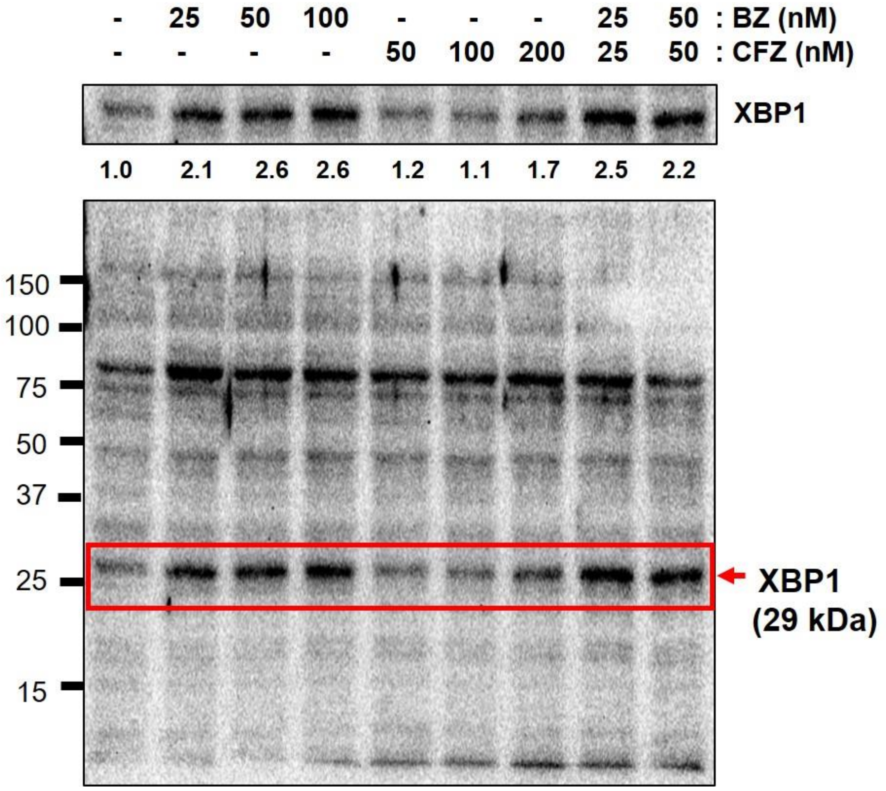
- 25 50 100 - - - 25 50 : BZ (nM)
- - - - 50 100 200 25 50 : CFZ (nM)
XBP1
1.0 2.1 2.6 2.6 1.2 1.1 1.7 2.5 2.2
150
100
75
50
37
25
15
XBP1
(29 kDa)

S9-8

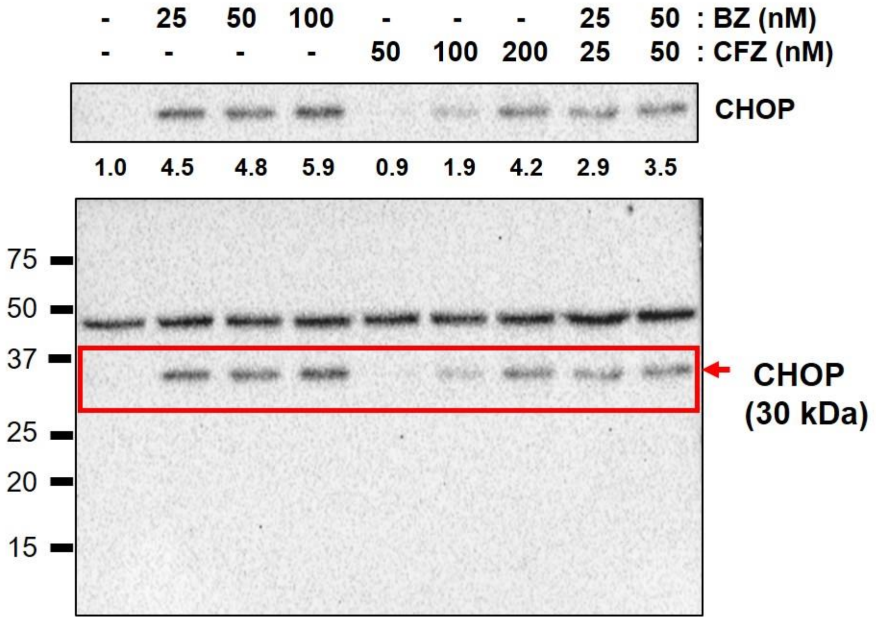
- 25 50 100 - - - 25 50 : BZ (nM)
- - - - 50 100 200 25 50 : CFZ (nM)
CHOP
1.0 4.5 4.8 5.9 0.9 1.9 4.2 2.9 3.5
75
50
37
25
20
15
CHOP
(30 kDa)

S9-9

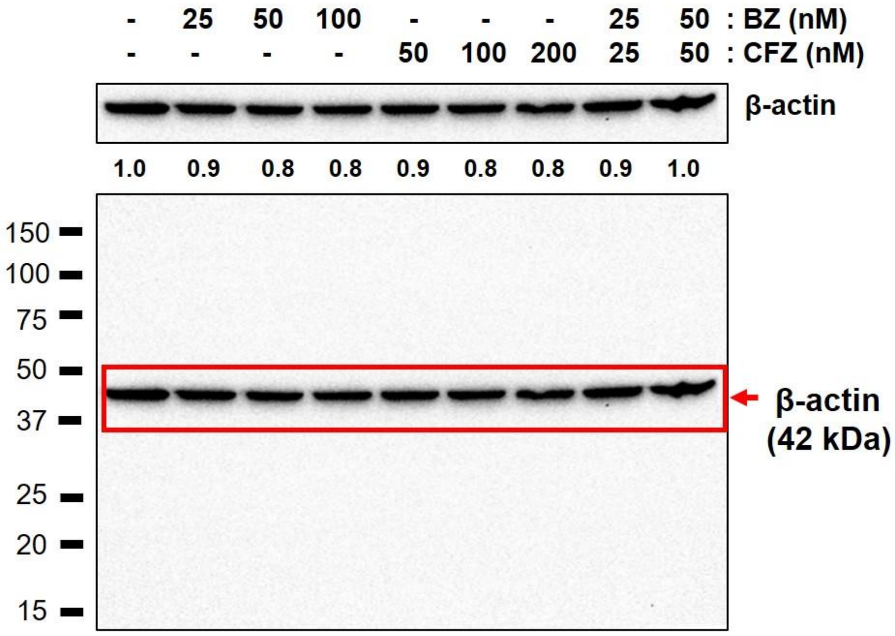

- 25 50 100 - - - 25 50 : BZ (nM)
- - - - 50 100 200 25 50 : CFZ (nM)
β-actin
1.0 0.9 0.8 0.8 0.9 0.8 0.8 0.9 1.0
150
100
75
50
37
25
20
15
β-actin
(42 kDa)
